# Rhodium(III) Complexes Featuring Coordinated CF_3_ Appendages

**DOI:** 10.1002/chem.201901184

**Published:** 2019-04-09

**Authors:** Jack Emerson‐King, Ivan Prokes, Adrian B. Chaplin

**Affiliations:** ^1^ Department of Chemistry University of Warwick Gibbet Hill Road Coventry CV4 7AL UK

**Keywords:** coordination chemistry, fluorinated ligands, low-coordinate complexes, phosphane ligands, rhodium

## Abstract

The synthesis and characterisation of a homologous series of rhodium 2,2′‐biphenyl complexes featuring intramolecular dative bonding of the nominally inert and weakly coordinating trifluoromethyl group are described. Presence of these interactions is evidenced in the solid state using X‐ray diffraction, with Rh−F contacts of 2.36–2.45 Å, and in solution using NMR spectroscopy, through hindered C−CF_3_ bond rotation and the presence of time‐averaged ^1^ *J*_RhF_ and ^2^ *J*_PF_ coupling.

The coordination chemistry of the transition elements is extensive, but notable for the paucity of well‐defined complexes featuring explicit C−F→M bonding interactions.^1^, ^2^ Indeed, the poor ligating characteristics of organofluorine groups, augmented by the inertness of the associated C−F bonds, lend them to notable application as constituents of weakly coordinating anions and solvents.^3^ Of the limited number of structurally characterised examples, the overwhelming majority are based on the electrophilic early transition metals: with **A**–**D** particularly notable (Figure 1).^4^, ^5^ Complexes of the platinum group metals are scarce and only **E**–**G** feature M−F contacts<2.5 Å.^6^, ^7^ Building on our recent work, employing the high *trans*‐influence 2,2′‐biphenyl (biph) ancillary ligand for the systematic study of agostic interactions,^8^ we herein report the synthesis and characterisation of an unprecedented homologous series of late transition metal complexes featuring distinct CF_3_→M bonding interactions.

**Figure 1:**
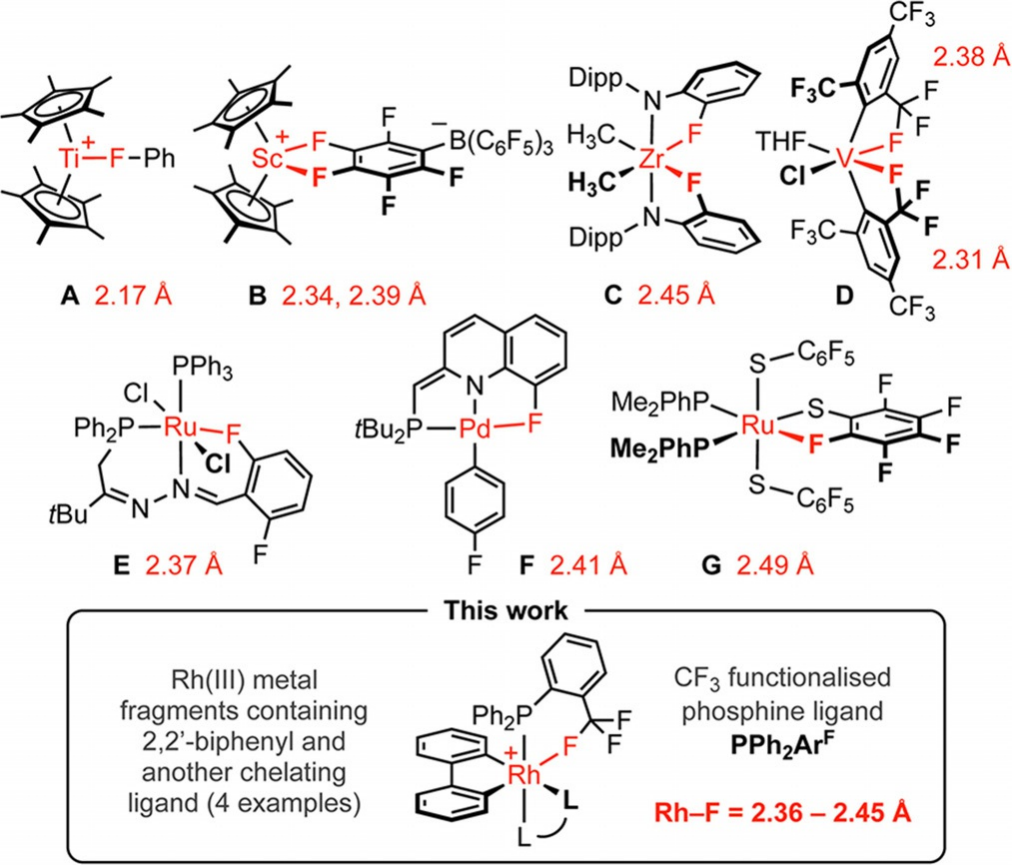
Selected examples of structurally characterised early and platinum group metal complexes featuring explicit C−F→M bonding interactions.

To temper the extremely low nucleophilicity of the CF_3_ group, we focused our efforts on probing the intramolecular coordination chemistry of this commonly used appendage and identified PPh_2_Ar^F^ as a prospective ditopic ligand (Figure 1).^9^ Monomeric Rh^III^ complex [Rh(biph)(dtbpm)Cl] (dtbpm=bis(di‐*tert*‐butylphosphino)methane) is an established source of the {Rh(biph)Cl} fragment in solution^8^, ^10^ and reaction with excess PPh_2_Ar^F^ in CH_2_Cl_2_ at RT proceeded, as anticipated, with substitution of the small bite‐angle diphosphine alongside precipitation of chloro‐bridged dinuclear complex **1** (Figure 2). The structure and purity of this sparingly soluble dimer was corroborated in (dilute) solution by NMR spectroscopy, in the solid state by single‐crystal X‐ray diffraction, and by combustion analysis. Subsequent substitution reactions enabled synthesis of considerably more soluble mononuclear derivatives **2**–**5**, which were all isolated in high purity and extensively characterised (Figure 2).

**Figure 2:**
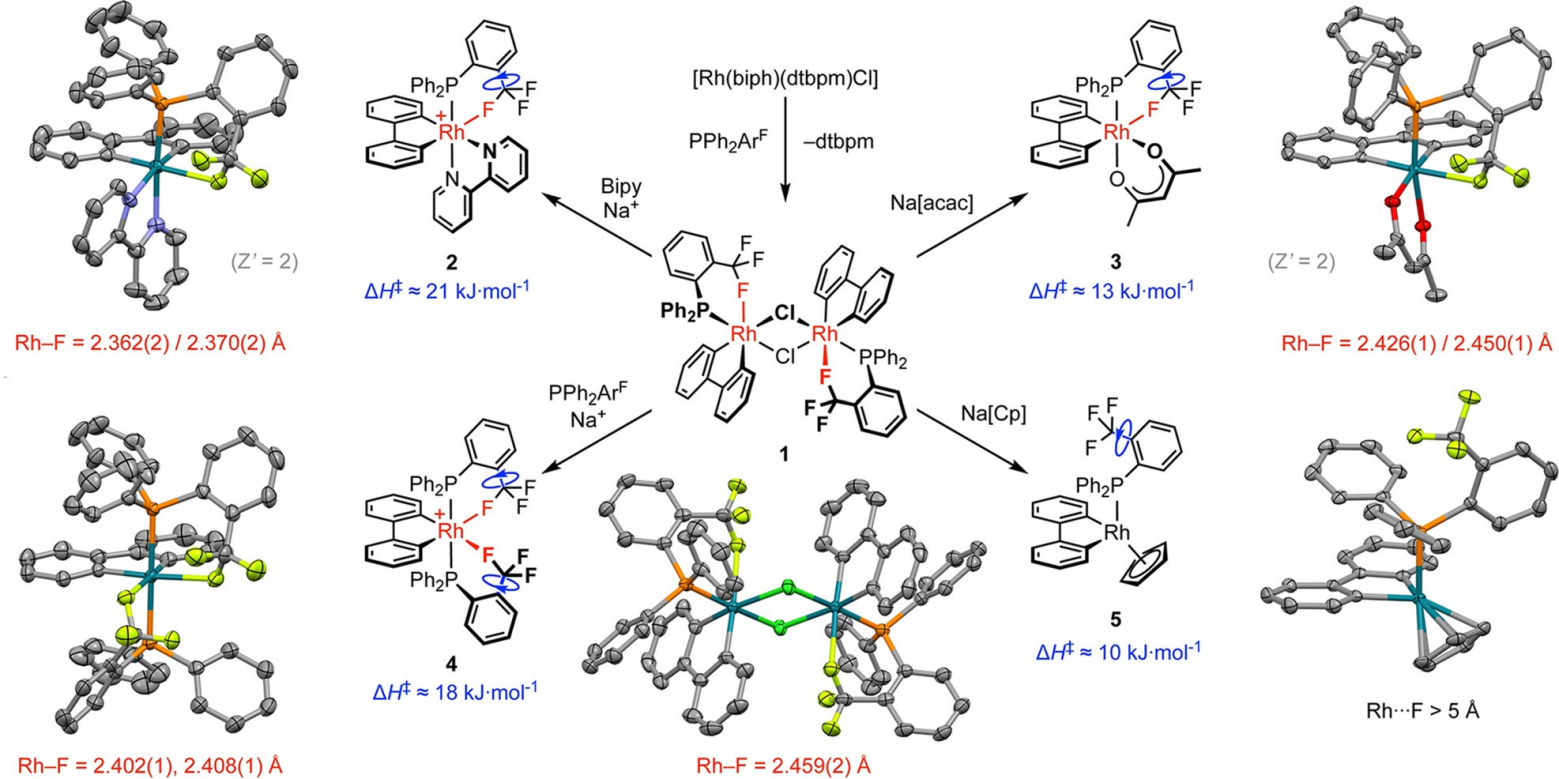
Synthesis, structures and dynamic properties of rhodium(III) complexes of PPh_2_Ar^F^; [B(3,5‐(CF_3_)_2_C_6_H_3_)_4_]^−^ counter anions omitted for clarity. All reactions were carried out in CH_2_Cl_2_ at RT; **1** was isolated in 82 % yield, and all subsequent substitution reactions proceeded quantitatively by NMR spectroscopy. Solid‐state structures drawn with thermal ellipsoids at 50 %, and minor disordered components (1×Ph group in **1** and **4**) and H atoms are omitted; symmetry equivalent atoms in **1** are generated by using the operation (4/3−*x*, 5/3−*y*, 2/3−*z*), only one of the two unique but structurally similar cations shown for **2** and **3** (*Z*′=2).^13^

The solid‐state structures of **1**–**4** are all notable for the adoption of distinct CF_3_→Rh bonding interactions, characterised by Rh−F contacts of 2.36–2.45 Å, increasing in the order **2**<**4**<**3**<**1**, and significant elongation of the bound C−F bond (ca. 0.04 Å). There are very few crystallographically characterised transition‐metal precedents for coordination of the CF_3_ appendage and, to the best of our knowledge,^1^, ^2^ only first‐row adduct **D** (Figure 1), bearing two rigid 2,4,6‐tris(trifluoromethyl)‐phenyl ligands, features a shorter contact [V−F=2.306(2) Å].^5^, ^11^ Coordination of cyclopentadienyl in **5** leads to the nominal monodentate coordination of PPh_2_Ar^F^, with the CF_3_ group projected away from the metal centre [∠Rh‐P‐C‐CCF_3_=167.7(1)° and Rh⋅⋅⋅F>5 Å] demonstrating that this phosphine ligand is sufficiently conformationally flexible as to not enforce the chelation observed in **1**–**4**.

In CD_2_Cl_2_ solution at 298 K, coordination of PPh_2_Ar^F^ in **1**–**5** was confirmed by ^31^P NMR spectroscopy with the associated resonances exhibiting large ^103^Rh coupling (^1^ *J*_RhP_=124–170 Hz). Further coupling to magnetically equivalent ^19^F nuclei (^2^ *J*_PF_≈5 Hz) is evident from the ^31^P{^1^H} NMR spectra of **1**–**4**, but absent in that of **5**, consistent with the presence of weak and time‐averaged CF_3_→Rh interactions in solution. At ambient temperature, fast rotation of the CF_3_ groups on the NMR time scale and coupling to both ^31^P and ^103^Rh, with ^1^ *J*_RhF_≈^2^ *J*_PF_ are also apparent from the ^19^F{^1^H} NMR spectra of **1**–**4** (*δ*_CF3_ −62.8 to −67.6 ppm; 376 MHz).^12^ The transient nature of the CF_3_→Rh interaction in solution inferred from these data is fully in line with expectation and further vindicated through pronounced structural dynamics of asymmetric **1**–**3** evident by ^1^H NMR spectroscopy at 298 K (400 MHz), that results in higher than expected time‐averaged symmetry of the biph ancillary ligand and invokes dissociation of the CF_3_ group. Equivalent exchange processes are presumably occurring in **4**, although the spectroscopic signatures are asymptomatic due to the inherently higher symmetry of this complex.

Further interrogation of **2**–**5** in CD_2_Cl_2_ was possible by variable‐temperature NMR spectroscopy (see Figure 3 and Supporting Information), with progressive cooling from 298 to 185 K freezing out the structural dynamics observed for **2** and **3** (^1^H NMR, 400 MHz), and inducing the onset of decoalescence of the CF_3_ resonances (^19^F NMR, 376 MHz). Although a full line shape analysis of the latter was not possible, as the slow exchange regime was not reached, the enthalpies of activation for hindered C−CF_3_ bond rotation could be estimated from the temperature dependence of the line width (Figures 2 and 3).^14^, ^15^ The activation barriers increase in the order **3**<**4**<**2**, correlating with the bond lengths observed in the solid state, and are all larger than that measured for **5**. Only minor broadening of the ^1^H Ar^F^ signals of **2**–**5** was observed on cooling, ruling out P−Ar^F^ bond rotation on the NMR time scale.

**Figure 3:**
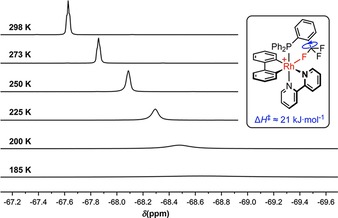
Variable‐temperature ^19^F{^1^H} NMR spectra of **2** (CD_2_Cl_2_, 376 MHz, 298–185 K).

Through the isolation and structural characterisation of Rh^III^ complexes of PPh_2_Ar^F^ **1**–**4** we have demonstrated the ability of the late‐transition‐metal complexes to form well‐defined, albeit weakly bound, adducts of the widely employed CF_3_ functional group. Synthesis of these complexes advances the coordination chemistry of weakly interacting organofluorine compounds, and highlights the use of C−F→M bonding interactions for the stabilisation of transition metal complexes with a low‐coordination number. On the basis of computational predictions,^16^ adducts of this nature have been predicted to be intermediates in the oxidative addition of C(sp^3^)−F bonds and our future work will be focused on testing this hypothesis experimentally.

## Conflict of interest

The authors declare no conflict of interest.

## Supporting information

As a service to our authors and readers, this journal provides supporting information supplied by the authors. Such materials are peer reviewed and may be re‐organized for online delivery, but are not copy‐edited or typeset. Technical support issues arising from supporting information (other than missing files) should be addressed to the authors.

Supplementary
