# Supplementary material for: Rhodium(III) Complexes Featuring Coordinated CF3 Appendages
Source: Chemistry. 2019 Apr 9;25(25):6317–9. doi: 10.1002/chem.201901184 (PMC6519252; doi:10.1002/chem.201901184)
Supplement: Supplementary file 1 — Supplementary [file CHEM-25-6317-s001.pdf]

CHEMISTRY
A European Journal

Supporting Information

Rhodium(III) Complexes Featuring Coordinated CF_3 Appendages

Jack Emerson-King, Ivan Prokes, and Adrian B. Chaplin*[a]

chem_201901184_sm_miscellaneous_information.pdf

Table of contents

1. General experimental methods S1
2. Preparation of [Rh(biph)(PPh$_2$Ar^F)Cl]$_2$ **1** S2
3. Preparation of [Rh(biph)(bipy)(PPh$_2$Ar^F)][BAr$^F_4$] **2** S5
4. Preparation of [Rh(biph)(acac)(PPh$_2$Ar^F)] **3** S9
5. Preparation of [Rh(biph)(PPh$_2$Ar^F)$_2$][BAr$^F_4$] **4** S13
6. Preparation of [Rh(biph)(Cp)(PPh$_2$Ar^F)] **5** S17
7. Line shape analysis S21
8. References S21

1. General experimental methods

All manipulations were performed under an atmosphere of argon using Schlenk and glove box techniques. Glassware was oven-dried at 150 °C overnight and flamed under vacuum prior to use. Anhydrous CH_2Cl_2 and hexane (<0.005% H_2O) were purchased from ACROS or Sigma-Aldrich and freeze–pump–thaw degassed three times before being placed under argon over thoroughly vacuum-dried 3 Å molecular sieves. CD_2Cl_2 was freeze–pump–thaw degassed three times before being placed under argon over thoroughly vacuum-dried 3 Å molecular sieves. [Rh(biph)(dtbpm)Cl],[1] PPh$_2$Ar^F,[2] Na[acac],[3] Na[Cp],[4] and Na[B(3,5-(CF$_3$)$_2$C$_6$H$_3$)$_4$][5] were synthesised using literature protocols; 2,2'-bipyridyl was purchased from Sigma-Aldrich and used as supplied. NMR spectra were recorded on either a Bruker Avance III HD 500 MHz or Bruker Avance III 400 MHz spectrometer (all variable temperature measurements). Chemical shirts are quoted in ppm and coupling constants in Hz. High-resolution electrospray ionisation mass spectra (HR ESI-MS) were recorded on a Bruker MaXis II spectrometer. Microanalysis was performed by Stephen Boyer at London Metropolitan University.

2. Preparation of $[Rh(biph)(PPh_2Ar^F)Cl]_2$ 1

A solution of [Rh(biph)(dtbpm)Cl] (297.5 mg, 0.500 mmol) and PPh_2Ar^F (412.9 mg, 1.250 mmol) in CH_2Cl_2 (3 mL) was stirred at ambient temperature for 18 hours. The resulting yellow microcrystalline precipitate was isolated by filtration, washed with CH_2Cl_2 (3 × 5 mL), and then dried *in vacuo* to afford the final product. Concentration of the combined filtrate and washings to *ca.* 5 mL afforded additional product on cooling to 4 °C, which was isolated by filtration, washed with CH_2Cl_2 (3 × 5 mL), and then dried *in vacuo*. Combined yield: 253.7 mg (82%, yellow solid).

^{1}H NMR (500 MHz, CD_2Cl_2, 298 K): δ 8.06 (dd, $^3J_{HH}$ = 8.0, $^3J_{PH}$ = 3.9, 2H, 6-Ar^F), 7.74 (t, $^3J_{HH}$ = 7.8, 2H, 5-Ar^F), 7.50 (t, $^3J_{HH}$ = 7.7, 2H, 4-Ar^F), 7.37 – 7.45 (m, 6H, 3-Ar^F + 6-biph), 7.20 (t, $^3J_{HH}$ = 7.5, 4H, *p*-Ph), 6.94 – 7.02 (m, 12H, 3-biph + *m*-Ph), 6.82 (t, $^3J_{HH}$ = 7.3, 4H, 4-biph), 6.64 (br, 8H, *o*-Ph), 6.62 (t, $^3J_{HH}$ = 7.5, 4H, 5-biph).

^{13}C{^{1}H} NMR (126 MHz, CD_2Cl_2, 298 K): δ 158.7 (HMBC, 1-biph), 151.5 (s, 2-biph), 137.3 (s, 3-Ar^F), 135.4 (s, 6-biph), 133.8 (d, $^2J_{PC}$ = 10, *o*-Ph), 134 (obscured, 2-Ar^F), 132.2 (d, $^4J_{PC}$ = 7, 4-Ar^F), 132.0 (s, 5-Ar^F), 130.6 (d, $^4J_{PC}$ = 2, *p*-Ph), 129.2 (d, $^1J_{PC}$ = 42, 1-Ar^F), 128.6 (br, 6-Ar^F), 128 (obscured, *i*-Ph), 127.8 (d, $^3J_{PC}$ = 11, *m*-Ph), 125.2 (s, 5-biph), 124.7 (q, $^1J_{FC}$ = 277, CF_3), 123.5 (s, 4-biph), 121.3 (s, 3-biph).

^{31}P{^{1}H} NMR (162 MHz, CD_2Cl_2, 298 K): δ 39.1 (dq, $^1J_{RhP}$ = 170, $^2J_{PF}$ = 4).

^{19}F{^{1}H} NMR (376 MHz, CD_2Cl_2, 298 K): δ -62.80 (vbr, fwhm = 15.5 Hz). Processing of this data using sine bell apodization resolved the broad CF_3 signal into an apparent triplet resonance with $^1J_{RhF} \approx {}^2J_{PF}$ = 3 Hz.

HR ESI-MS (positive ion, 4 kV): 626.0718 ([½M–Cl+MeCN]$^+$, calcd 626.0726) *m/z*.

Anal. Calcd for $C_{62}H_{44}Cl_2F_6P_2Rh_2$ (1241.68 g·mol^{-1}): C, 59.97; H, 3.57; N, 0.00. Found: C, 59.90; H, 3.64; N, 0.0.

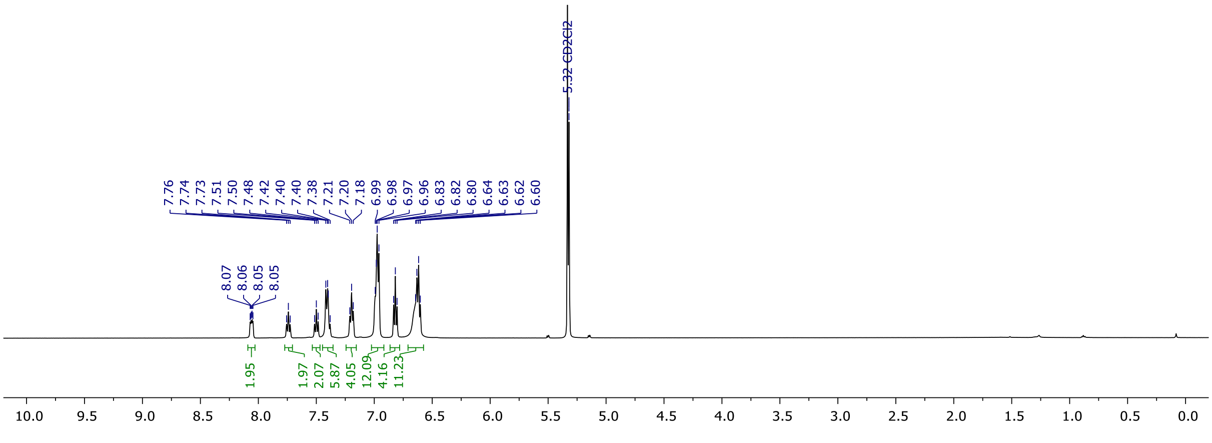

Figure S1: ^{1}H NMR spectrum of $[Rh(biph)(PPh_2Ar^F)Cl]_2$ **1** (500 MHz, CD_2Cl_2, 298 K).

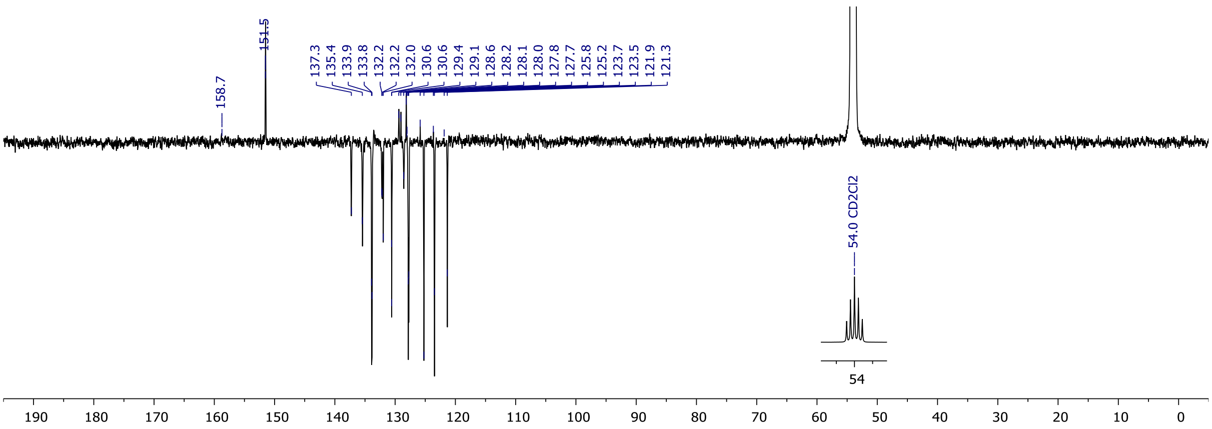

Figure S2: $^{13}C\{^{1}H\}$ APT NMR spectrum of $[Rh(biph)(PPh_2Ar^F)Cl]_2$ **1** (126 MHz, CD_2Cl_2, 298 K).

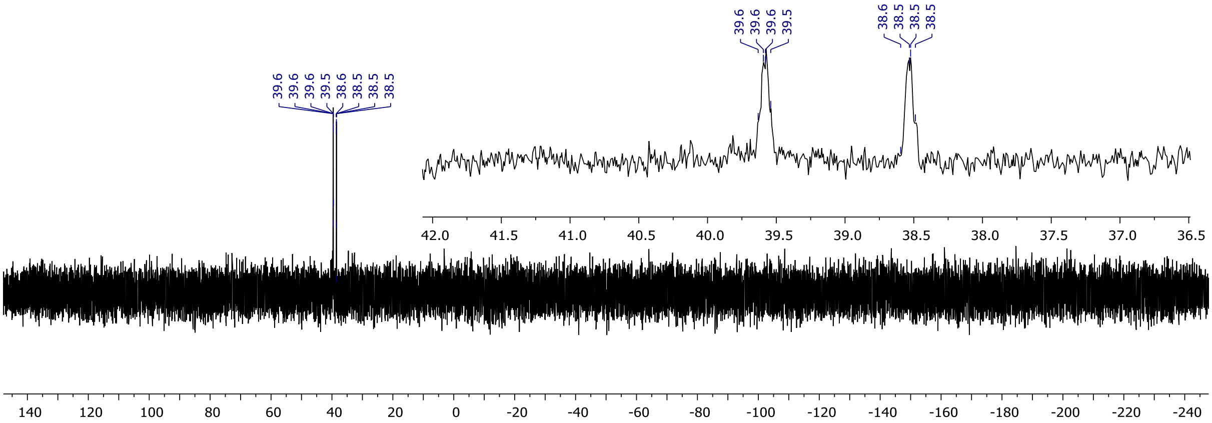

Figure S3: $^{31}P\{^{1}H\}$ NMR spectrum of $[Rh(biph)(PPh_2Ar^F)Cl]_2$ **1** (162 MHz, CD_2Cl_2, 298 K).

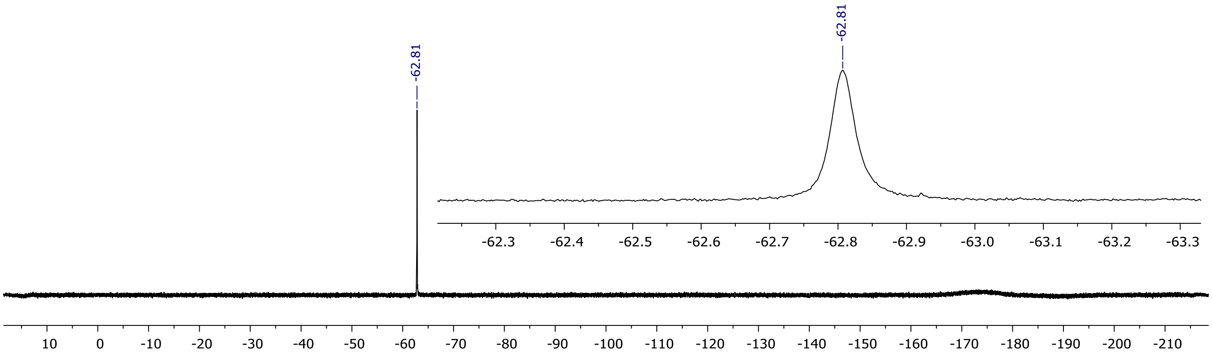

Figure S4: $^{19}F\{^{1}H\}$ NMR spectrum of $[Rh(biph)(PPh_2Ar^F)Cl]_2$ **1** (376 MHz, CD_2Cl_2, 298 K).

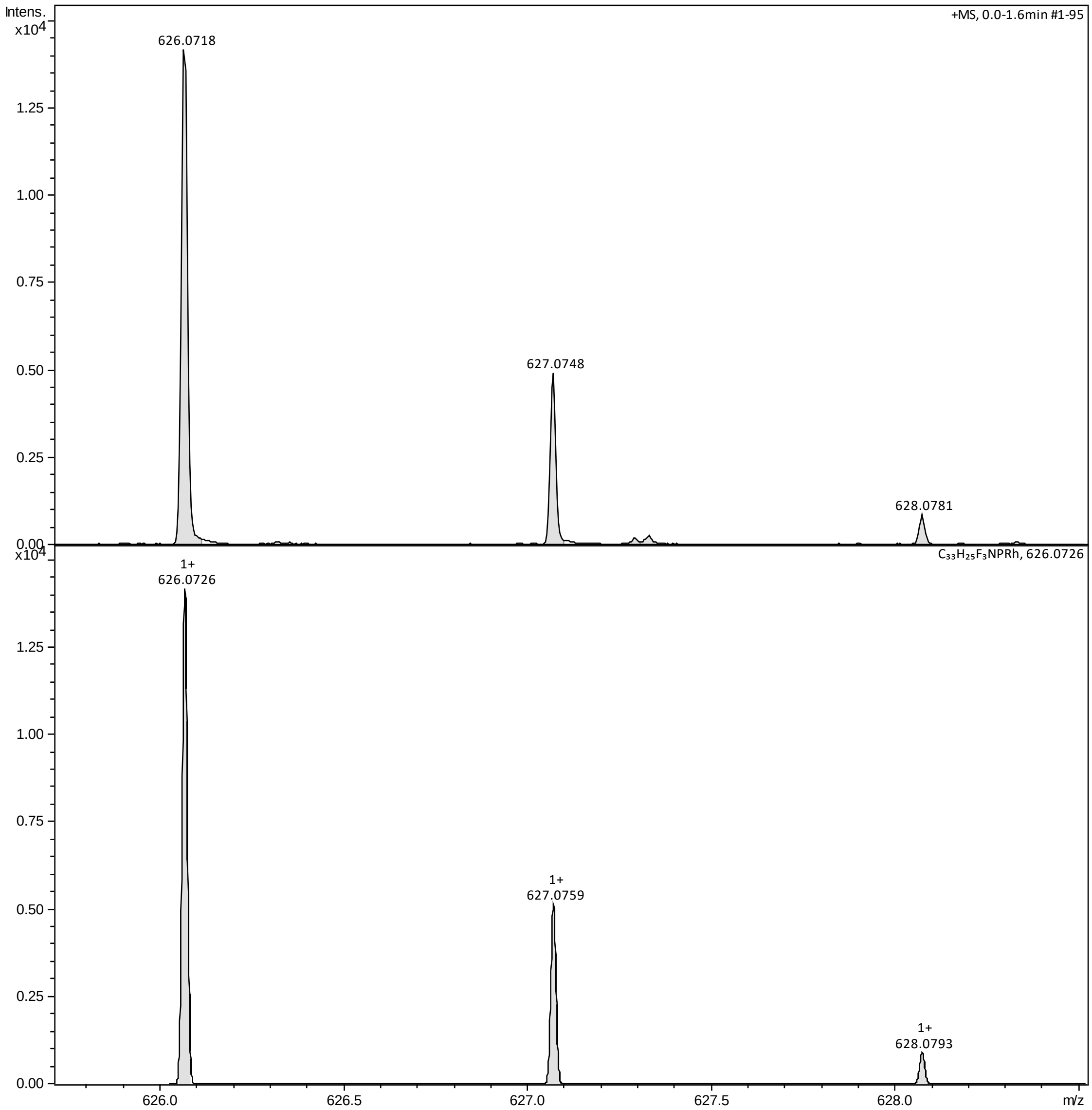

Figure S5: HR ESI-MS spectrum of $[Rh(biph)(PPh_2Ar^F)Cl]_2$ **1**.

3. Preparation of $[Rh(biph)(bipy)(PPh_2Ar^F)][BAr^F_4]$ 2

A suspension of **1** (62.1 mg, 50.0 mmol), $Na[B(3,5-(CF_3)_2C_6H_3)_4]$ (97.5 mg, 110 µmol), and 2,2'-bipyridyl (17.2 mg, 110 µmol) in CH_2Cl_2 (5 mL) was stirred at ambient temperature for 18 h. The resulting yellow solution was filtered, and the product crystallised by the addition of excess hexane (*ca*. 20 mL). Yield: 137.2 g (85%, yellow solid).

^{1}H NMR (500 MHz, CD_2Cl_2, 298 K): δ 8.24 (d, $^3J_{HH}$ = 8.1, 1H, 3'-bipy), 8.07 – 8.14 (m, 3H, 6-Ar^F + 3-bipy + 4'-bipy), 7.90 (t, $^3J_{HH}$ = 7.8, 1H, 4-bipy), 7.87 (t, $^3J_{HH}$ = 7.8, 1H, 5-Ar^F), 7.75 – 7.78 (m, 1H, 6-bipy), 7.70 – 7.75 (m, 8H, $[B(3,5-(CF_3)_2C_6H_3)_4]^-$), 7.63 – 7.69 (m, 2H, 4-Ar^F + 6'-bipy), 7.55 (br, 4H, $[B(3,5-(CF_3)_2C_6H_3)_4]^-$), 7.41 (dd, $^3J_{PH}$ = 10.9, $^3J_{HH}$ = 8.0, 1H, 3-Ar^F), 7.27 (t, $^3J_{HH}$ = 6.6, 1H, 5'-bipy), 7.20 (t, $^3J_{HH}$ = 6.7, 1H, 5-bipy), 5.80 – 8.00 (br m, 18 H, biph + Ph).

^{13}C{^{1}H} NMR (126 MHz, CD_2Cl_2, 298 K): δ 162.3 (q, $^1J_{CB}$ = 50, $[B(3,5-(CF_3)_2\underline{C}_6H_3)_4]^-$), 155.94 (d, $^2J_{RhC}$ = 2, 2/2'-bipy), 155.92 (d, $^2J_{RhC}$ = 1, 2'/2-bipy), 152.9 (d, $^2J_{RhC}$ = 2, 6'-bipy), 151.7 (app. t, $^2J_{RhC} \approx {}^3J_{PC}$ = 2, 6-bipy), 151.3 (br, tentatively assigned to 1-biph), 140.7 (s, 4'-bipy), 140.5 (s, 4-bipy), 138.5 (d, $^3J_{PC}$ = 2, 3-Ar^F), 135.4 (s, $[B(3,5-(CF_3)_2\underline{C}_6H_3)_4]^-$), 134.1 (br, tentatively assigned to *o*-Ph), 133.7 (d, $^4J_{PC}$ = 7, 4-Ar^F), 133.2 (d, $^3J_{PC}$ = 2, 5-Ar^F), 132.8 (qd, $^2J_{FC}$ = 29, $^2J_{PC}$ = 11, 2-Ar^F), 129.4 (qq, $^2J_{FC}$ = 32, $^3J_{CB}$ = 3, $[B(3,5-(CF_3)_2\underline{C}_6H_3)_4]^-$), 128.8 (app p, $^4J_{FC} \approx {}^2J_{PC}$ = 6, 6-Ar^F), 127.59 (br, 5-bipy), 127.57 (d, $^1J_{PC}$ = 42, 1-Ar^F), 127.0 (s, 5'-bipy), 126.9 (s, tentatively assigned to *p*-Ph), 125.5 (qd, $^1J_{FC}$ = 275, $^3J_{PC}$ = 2, CF_3), 125.3 (br, tentatively assigned to *m*-Ph), 125.2 (q, $^1J_{FC}$ = 272, $[B(3,5-(\underline{C}F_3)_2C_6H_3)_4]^-$), 123.4 – 123.5 (m, 3-bipy + 3'-bipy), 118.0 (sept, $^3J_{FC}$ = 4, $[B(3,5-(CF_3)_2\underline{C}_6H_3)_4]^-$). Due to structural dynamics on the NMR time scale the signals for biph and Ph are either not observed or not unambiguously assigned.

^{31}P{^{1}H} NMR (162 MHz, CD_2Cl_2, 298 K): δ 32.6 (dq, $^1J_{RhP}$ = 150, $^2J_{PF}$ = 5).

^{19}F{^{1}H} NMR (376 MHz, CD_2Cl_2, 298 K): δ -62.89 (s, $[B(3,5-(CF_3)_2C_6H_3)_4]^-$), -67.61 (app t, $^1J_{RhF} \approx {}^2J_{RhP}$ = 4.0, Ar^F).

HR ESI-MS (positive ion, 4 kV): 741.1144 ($[M]^+$, calcd 741.1148) *m*/*z*.

Anal. Calcd for $C_{73}H_{42}BF_{27}N_2PRh$ (1604.80 g·mol^{-1}): C, 54.64; H, 2.64; N, 1.75. Found: C, 54.49; H, 2.80; N, 1.74.

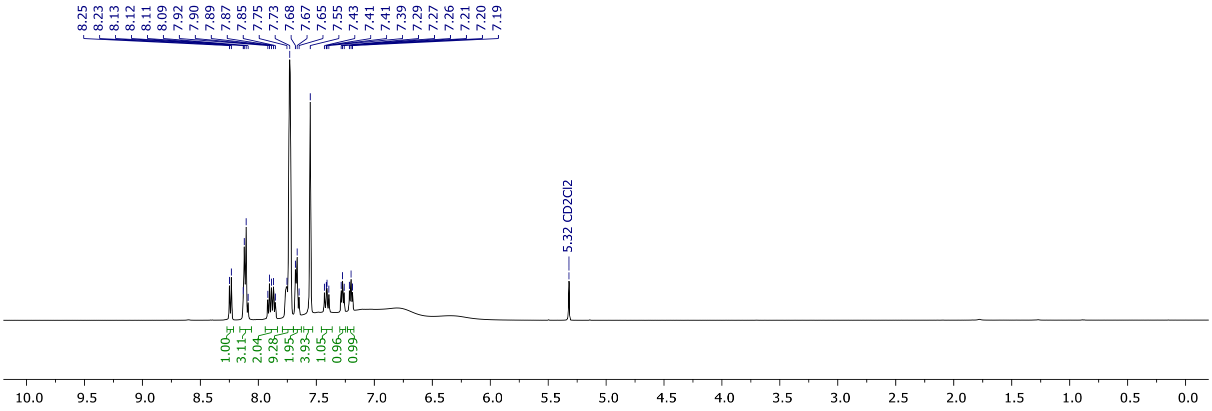

Figure S6: ^{1}H NMR spectrum of [Rh(biph)(bipy)(PPh$_2$Ar^F)][BAr$^F_4$] **2** (500 MHz, CD_2Cl_2, 298 K).

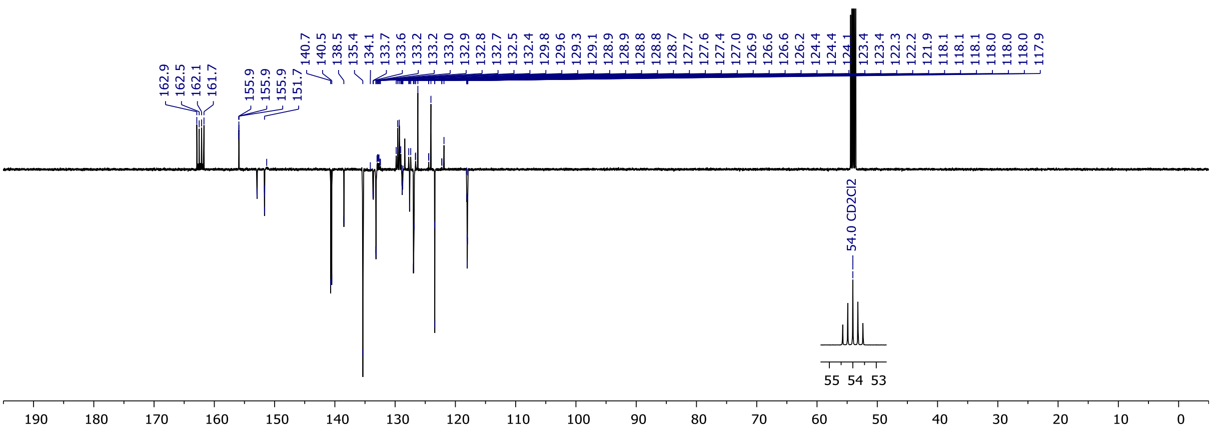

Figure S7: $^{13}C\{^{1}H\}$ APT NMR spectrum of [Rh(biph)(bipy)(PPh$_2$Ar^F)][BAr$^F_4$] **2** (126 MHz, CD_2Cl_2, 298 K).

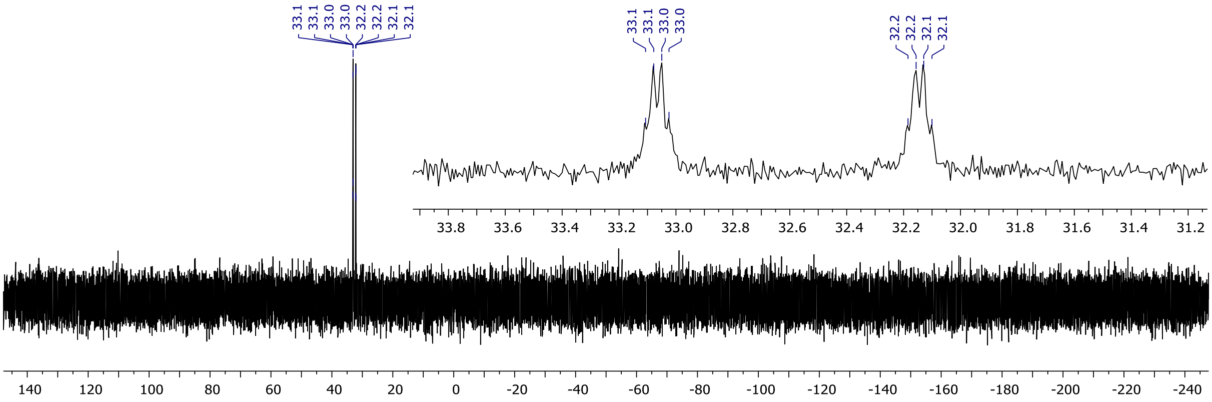

Figure S8: $^{31}P\{^{1}H\}$ NMR spectrum of [Rh(biph)(bipy)(PPh$_2$Ar^F)][BAr$^F_4$] **2** (162 MHz, CD_2Cl_2, 298 K).

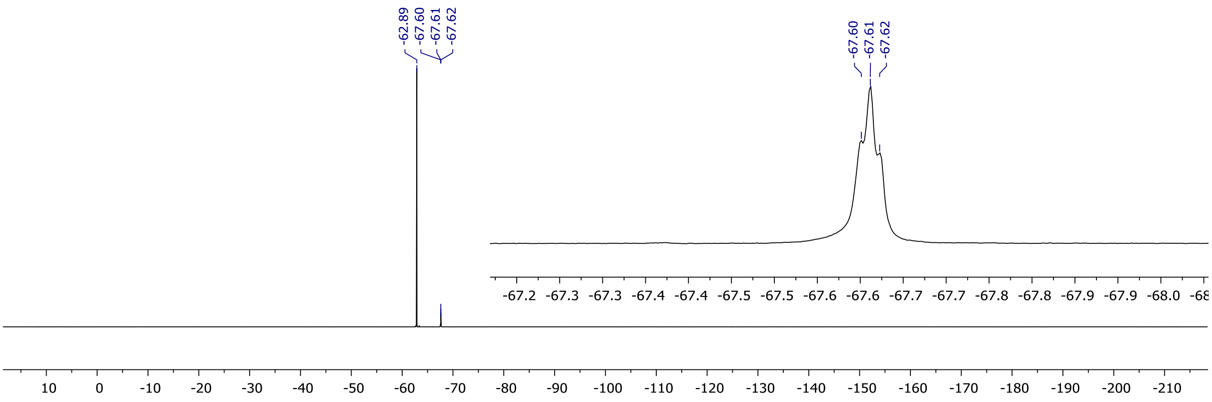

Figure S9: $^{19}F\{^1H\}$ NMR spectra of $[Rh(biph)(bipy)(PPh_2Ar^F)][BAr^F_4]$ **2** (376 MHz, CD_2Cl_2, 298 K).

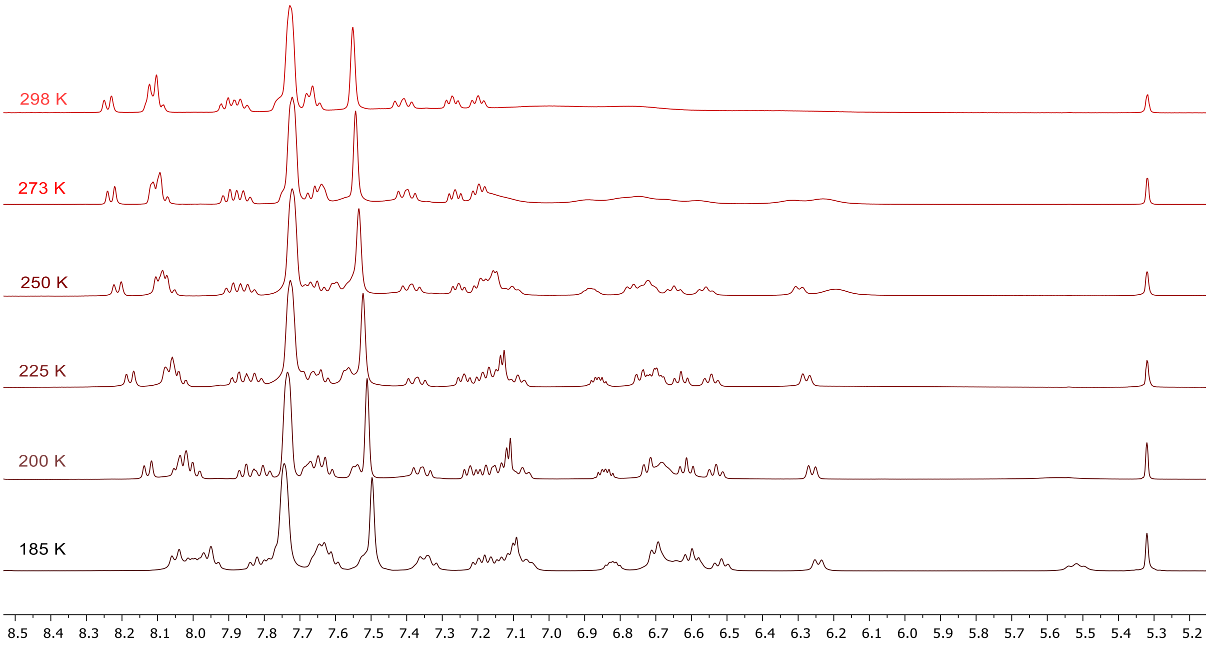

Figure S10: VT ^1H NMR spectra of $[Rh(biph)(bipy)(PPh_2Ar^F)][BAr^F_4]$ **2** (400 MHz, CD_2Cl_2).

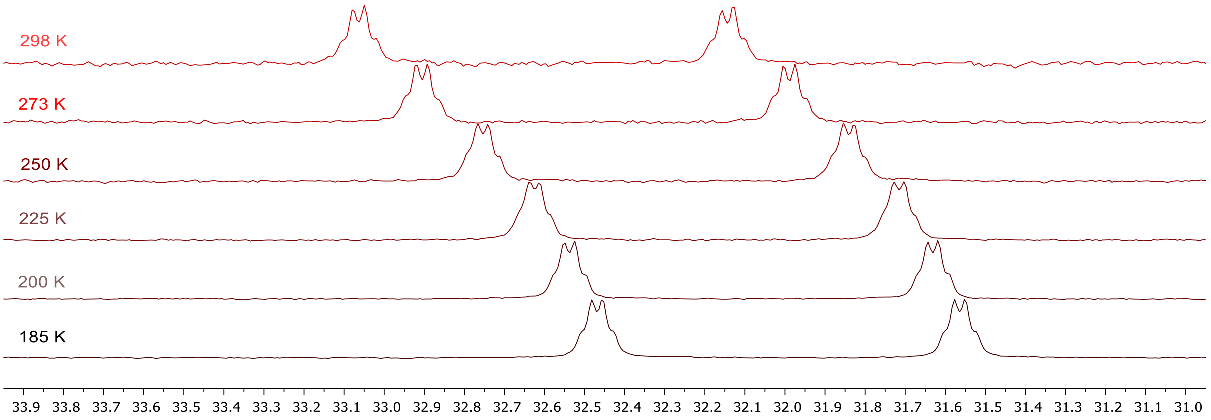

Figure S11: VT $^{31}P\{^1H\}$ NMR spectra of $[Rh(biph)(bipy)(PPh_2Ar^F)][BAr^F_4]$ **2** (162 MHz, CD_2Cl_2).

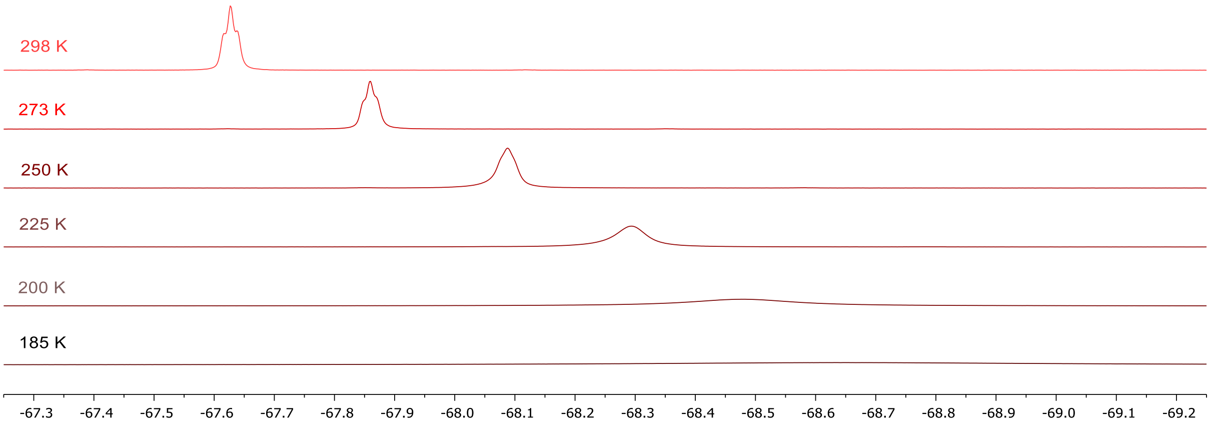

Figure S12: VT $^{19}F\{^1H\}$ NMR spectra of $[Rh(biph)(bipy)(PPh_2Ar^F)][BAr^F{}_4]$ **2** (376 MHz, CD_2Cl_2).

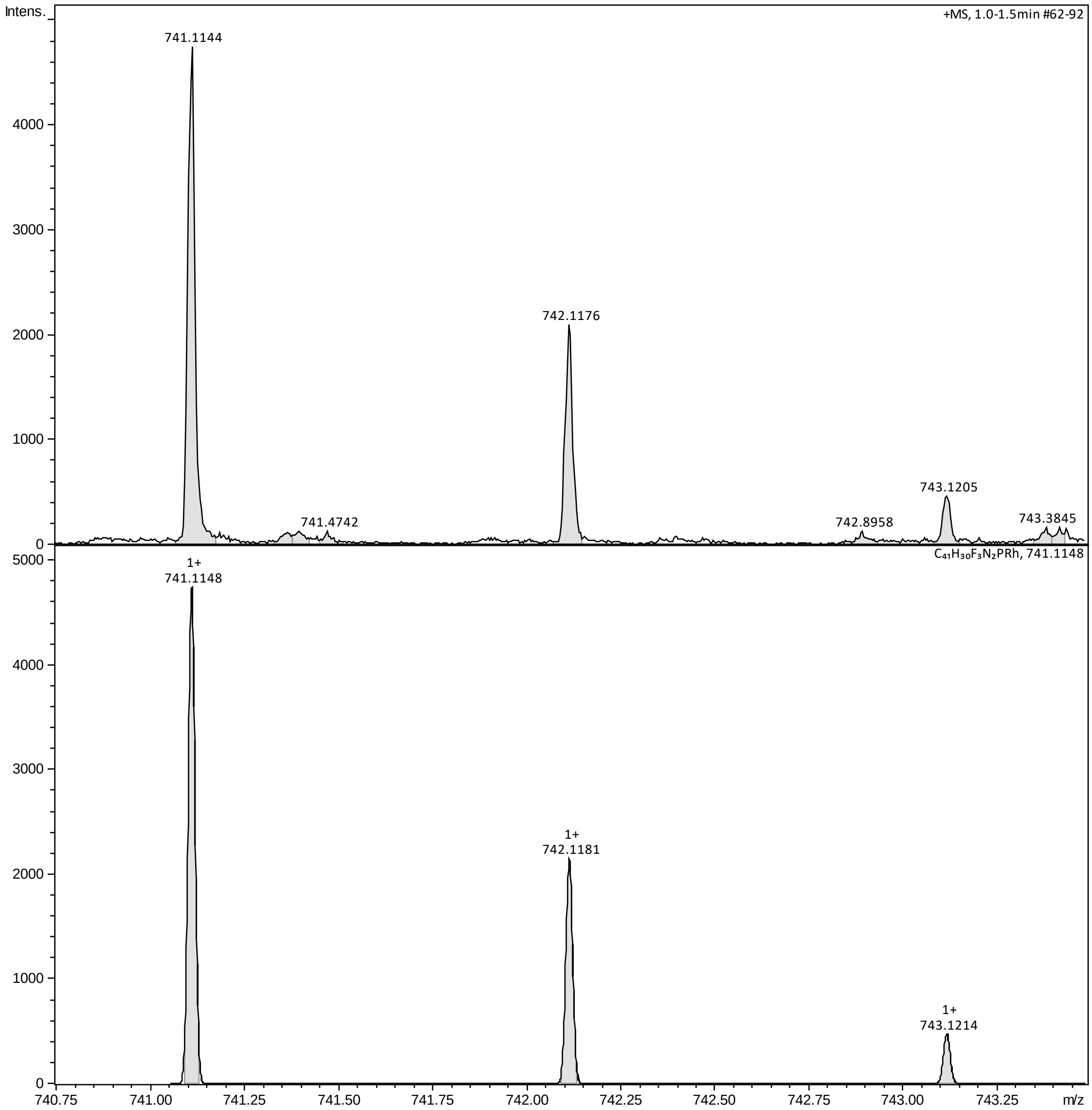

Figure S13: HR ESI-MS spectrum of $[Rh(biph)(bipy)(PPh_2Ar^F)][BAr^F{}_4]$ **2**.

4. Preparation of [Rh(biph)(acac)(PPh_2Ar^F)] 3

A suspension of **1** (62.1 mg, 50.0 mmol), Na[acac] (13.4 mg, 110 µmol) in CH_2Cl_2 (5 mL) was stirred at ambient temperature for 18 h. The resulting yellow solution was filtered, and the product crystallised by the addition of excess hexane (*ca*. 20 mL). Yield: 48.4 mg (75%, yellow solid).

^{1}H NMR (500 MHz, CD_2Cl_2, 298 K): δ 8.03 (dd, $^3J_{HH}$ = 8.0, $^3J_{PH}$ = 4.1, 1H, 6-Ar^F), 7.72 (t, $^3J_{HH}$ = 7.6, 1H, 5-Ar^F), 7.55 (t, $^3J_{HH}$ = 7.6, 1H, 4-Ar^F), 7.50 (t, $^3J_{HH}$ = 8.8, 1H, 3-Ar^F), 7.25 (t, $^3J_{HH}$ = 7.3, 2H, *p*-Ph), 7.01 – 7.08 (m, 6H, 6-biph + *m*-Ph), 6.96 (dd, $^3J_{HH}$ = 7.5, $^4J_{HH}$ = 1.6, 2H, 3-biph), 6.88 (app. t, $^3J_{HH} \approx {}^3J_{PH}$ = 10, 4H, *o*-Ph), 6.79 (t, $^3J_{HH}$ = 7.4, 2H, 4-biph), 6.60 (td, $^3J_{HH}$ = 7.5, $^4J_{HH}$ = 1.6, 2H, 5-biph), 5.43 (s, 1H, CH), 1.94 (s, 6H, CH_3).

^{13}C{^{1}H} NMR (126 MHz, CD_2Cl_2, 298 K): δ 187.5 (s, CO), 160.8 (br d, $^1J_{RhC}$ = 37, 1-biph), 152.4 (s, 2-biph), 135.8 (s, 3-Ar^F), 134.5 (d, $^2J_{PC}$ = 10, *o*-Ph), 134.2 (s, 6-biph), 132.7 (qd, $^2J_{FC}$ = 30, $^2J_{PC}$ = 11, 2-Ar^F), 132.3 (d, $^4J_{PC}$ = 6, 4-Ar^F), 131.5 (d, $^3J_{PC}$ = 2, 5-Ar^F), 131.1 (d, $^1J_{PC}$ = 40, 1-Ar^F), 130.4 (d, $^4J_{PC}$ = 2, *p*-Ph), 129.0 (d, $^1J_{PC}$ = 54, *i*-Ph), 128.5 (app. p, $^2J_{PC} \approx {}^4J_{FC}$ = 7, 6-Ar^F), 127.6 (d, $^3J_{PC}$ = 11, *m*-Ph), 125.2 (qd, $^1J_{FC}$ = 275, $^3J_{PC}$ = 2, CF_3), 124.9 (s, 5-biph), 123.4 (s, 4-biph), 121.2 (s, 3-biph), 99.2 (s, CH), 28.4 (s, CH_3), 28.4 (s, CH_3).

^{31}P{^{1}H} NMR (162 MHz, CD_2Cl_2, 298 K): δ 38.9 (dq, $^1J_{RhP}$ = 162, $^2J_{PF}$ = 6).

^{19}F{^{1}H} NMR (376 MHz, CD_2Cl_2, 298 K): δ -64.13 (app. t, $^1J_{RhF} \approx {}^2J_{PF}$ = 5).

HR ESI-MS (positive ion, 4 kV): 684.0905 ($[M]^{+\cdot}$, calcd 684.0907) *m/z*.

Anal. Calcd for $C_{36}H_{29}F_3O_2PRh$ (684.50 g·mol^{-1}): C, 63.17; H, 4.27; N, 0.00. Found: C, 63.10; H, 4.25; N, 0.00.

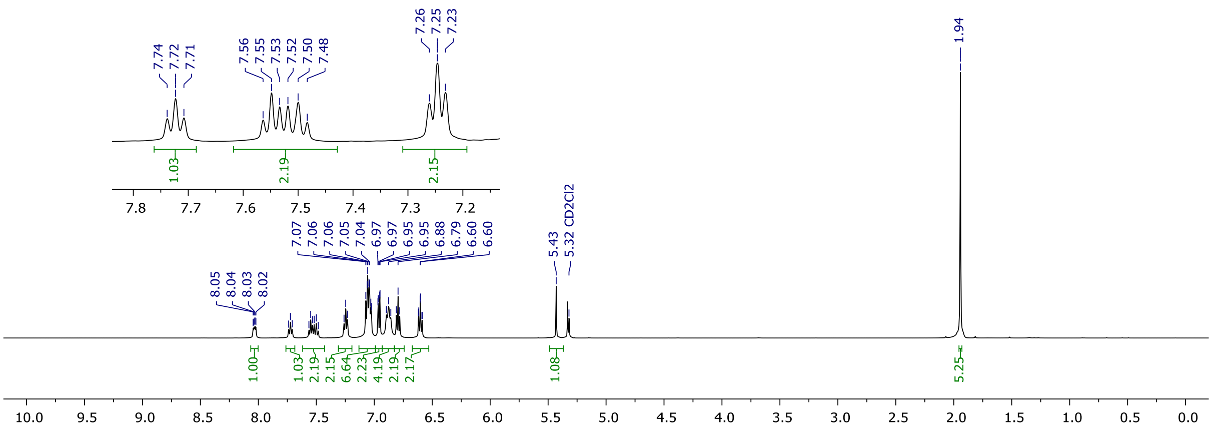

Figure S14: ^{1}H NMR spectrum of [Rh(biph)(acac)(PPh_2Ar^F)] **3** (500 MHz, CD_2Cl_2, 298 K).

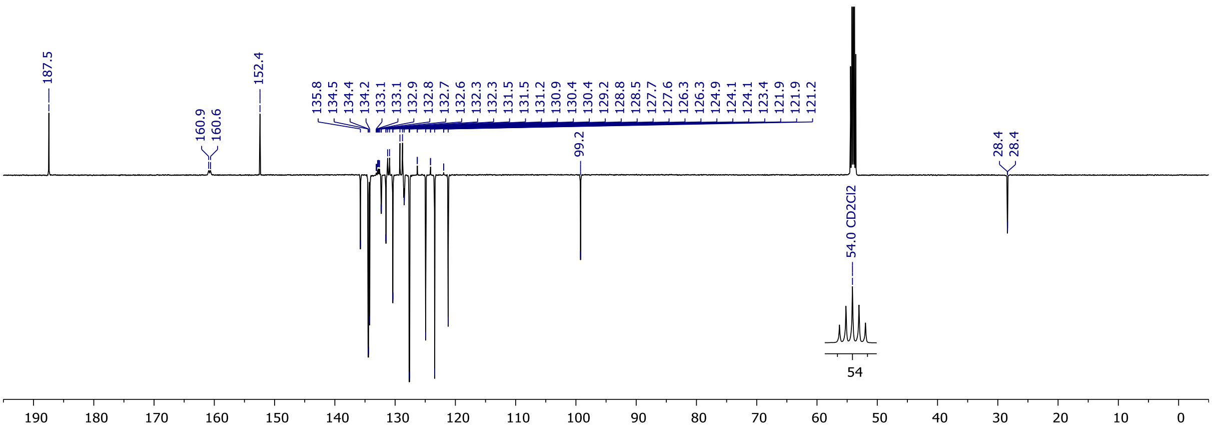

Figure S15: $^{13}C\{^{1}H\}$ APT NMR spectrum of [Rh(biph)(acac)(PPh_2Ar^F)] **3** (126 MHz, CD_2Cl_2, 298 K).

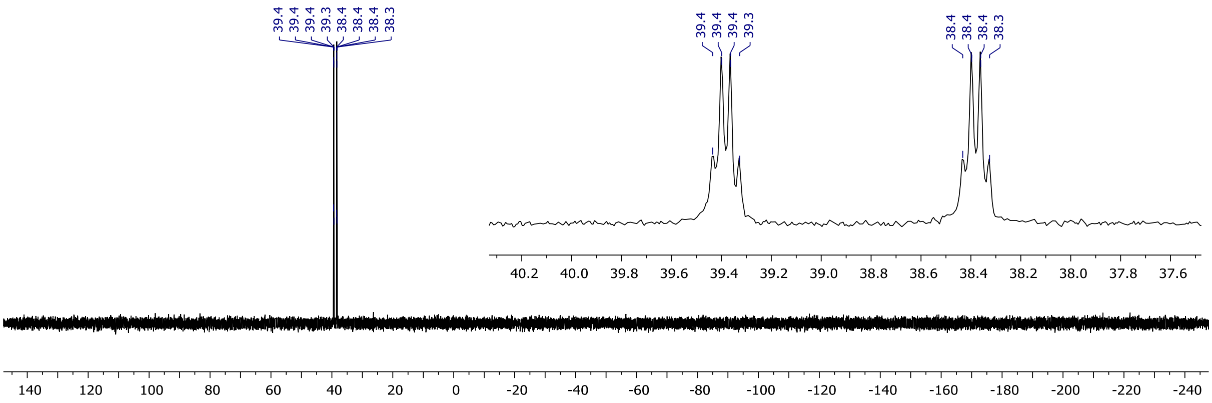

Figure S16: $^{31}P\{^{1}H\}$ NMR spectrum of [Rh(biph)(acac)(PPh_2Ar^F)] **3** (162 MHz, CD_2Cl_2, 298 K).

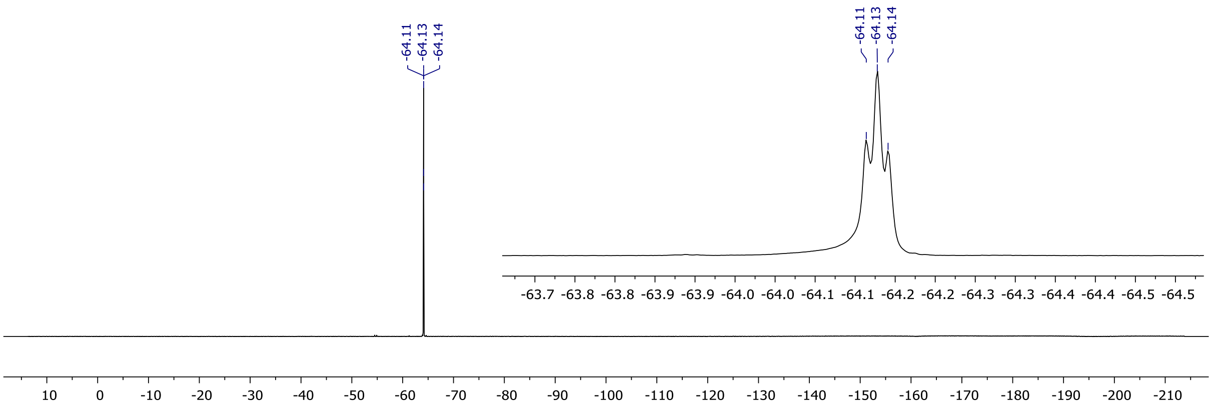

Figure S17: $^{19}F\{^{1}H\}$ NMR spectrum of [Rh(biph)(acac)(PPh_2Ar^F)] **3** (376 MHz, CD_2Cl_2, 298 K).

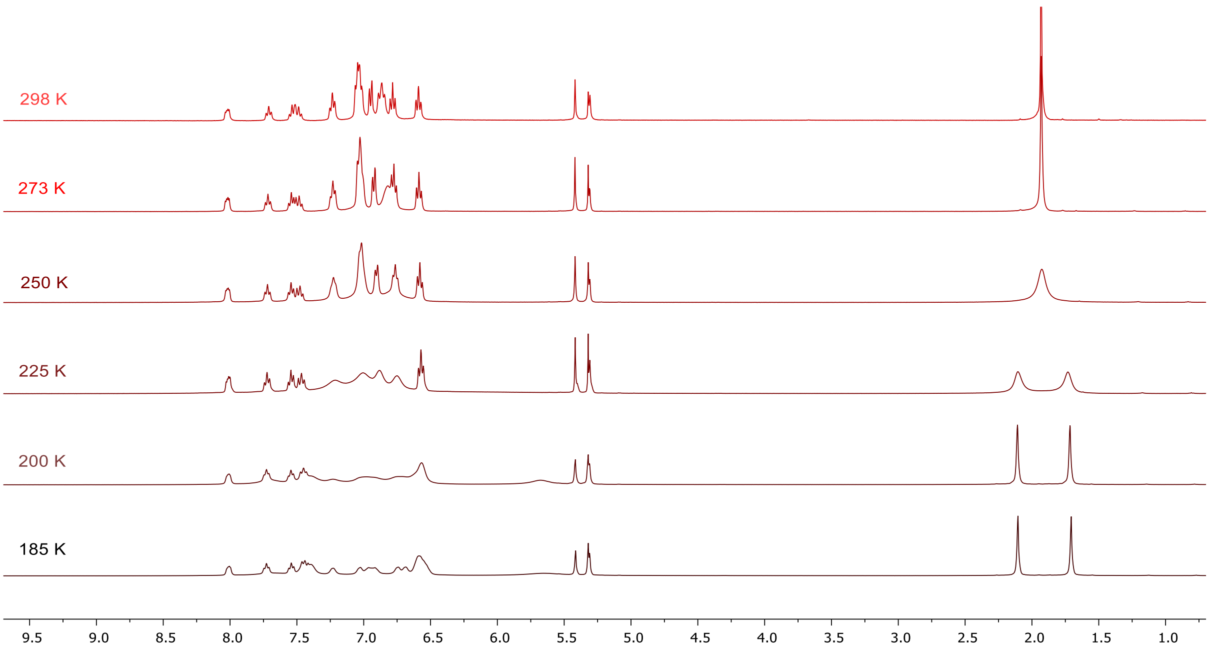

Figure S18: VT ^{1}H NMR spectra of [Rh(biph)(acac)(PPh_2Ar^F)] **3** (400 MHz, CD_2Cl_2).

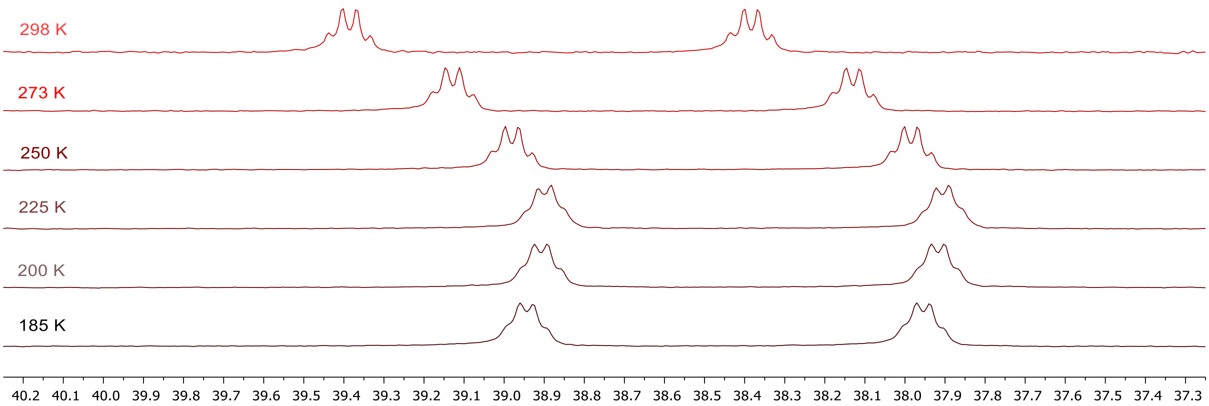

Figure S19: VT $^{31}P\{^{1}H\}$ NMR spectra of [Rh(biph)(acac)(PPh_2Ar^F)] **3** (162 MHz, CD_2Cl_2).

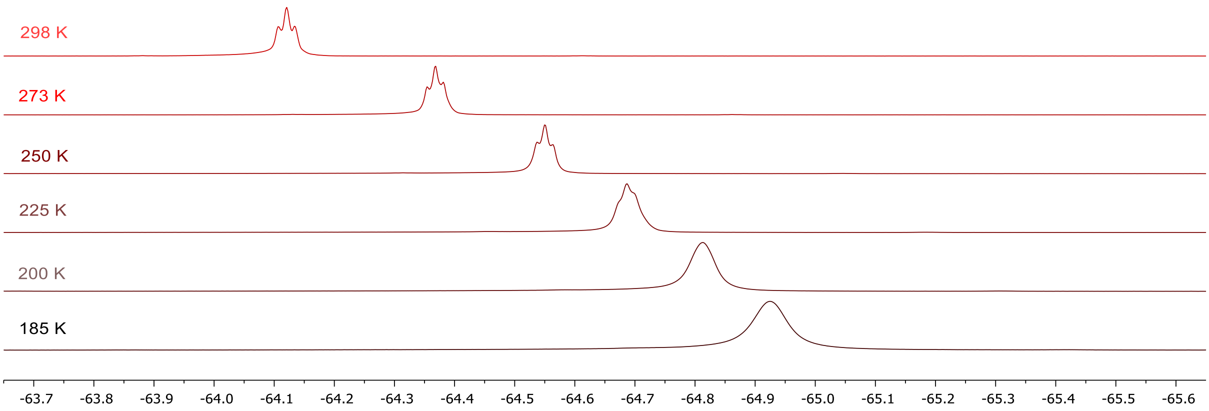

Figure S20: VT $^{19}F\{^{1}H\}$ NMR spectra of [Rh(biph)(acac)(PPh_2Ar^F)] **3** (376 MHz, CD_2Cl_2).

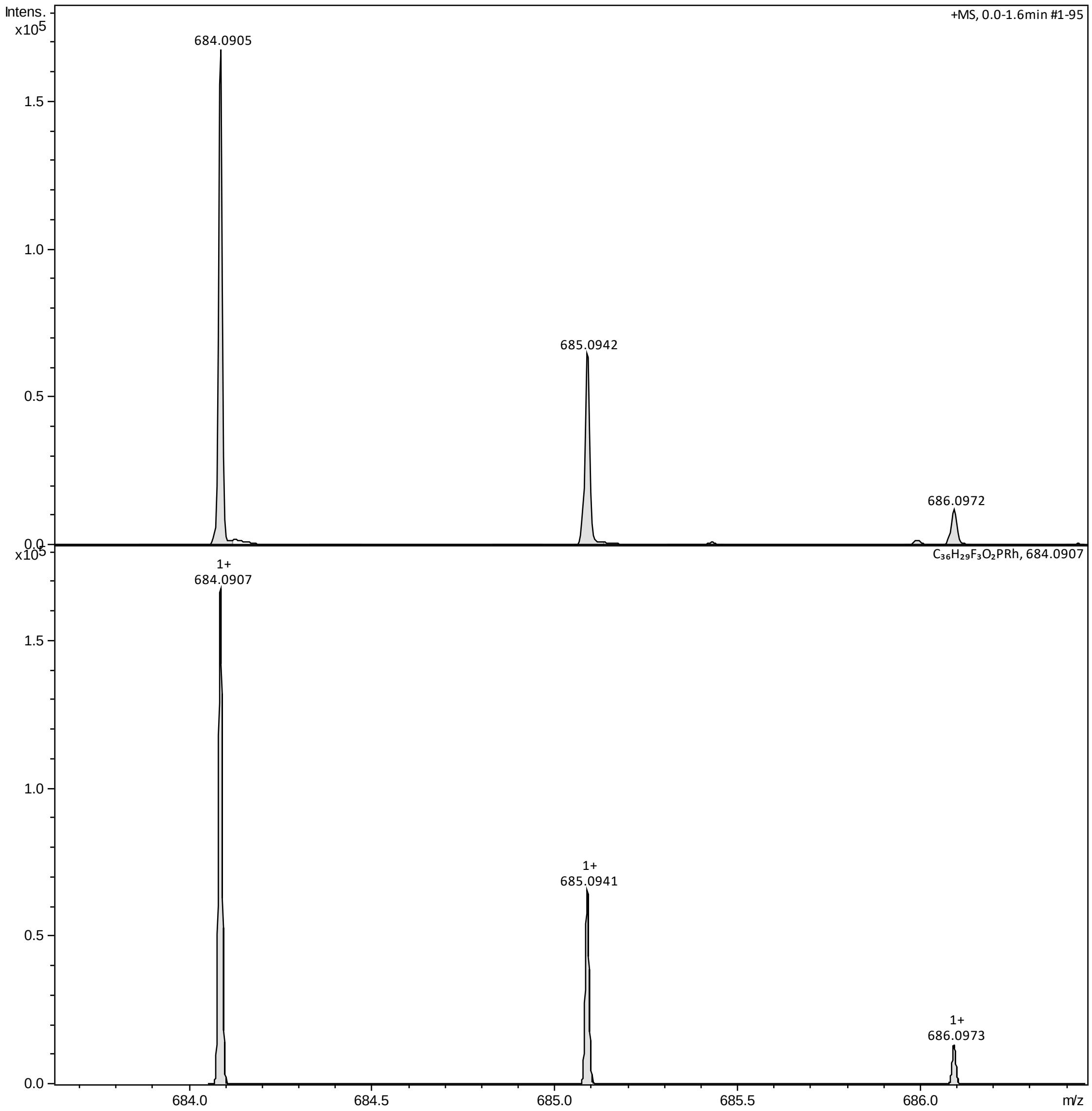

Figure S21: HR ESI-MS spectrum of [Rh(biph)(acac)(PPh_2Ar^F)] **3.**

5. Preparation of [Rh(biph)(PPh_2Ar^F)$_2$][BAr^F_4] 4

A suspension of **1** (62.1 mg, 50.0 µmol), Na[B(3,5-(CF_3)$_2C_6H_3$)$_4$] (97.5 mg, 110 µmol), and PPh_2Ar^F (36.3 mg, 110 µmol) in CH_2Cl_2 (5 mL) was stirred at ambient temperature for 18 h. The resulting pale-yellow solution was filtered, and the product crystallised by the addition of excess hexane (*ca*. 20 mL). Yield: 153.9 mg (87%, pale yellow solid).

^{1}H NMR (500 MHz, CD_2Cl_2, 298 K): δ 8.13 (d, $^3J_{HH}$ = 7.8, 2H, 6-Ar^F), 7.87 (t, $^3J_{HH}$ = 7.8, 2H, 5-Ar^F), 7.75 – 7.70 (m, 8H, [B(3,5-(CF_3)$_2C_6H_3$)$_4$]$^-$), 7.69 (t, $^3J_{HH}$ = 7.8, 2H, 4-Ar^F), 7.55 (br, 4H, [B(3,5-(CF_3)$_2C_6H_3$)$_4$]$^-$), 7.49 (dt, $^3J_{HH}$ = 7.8, $^3J_{PH}$ = 5.0, 2H, 5-Ar^F), 7.36 (t, $^3J_{HH}$ = 7.5, 4H, *p*-Ph), 7.10 (t, $^3J_{HH}$ = 7.8, 8H, m-Ph), 7.03 (d, $^3J_{HH}$ = 8.0, 2H, 6-biph), 6.78 (t, $^3J_{HH}$ = 7.4, 2H, 4-biph), 6.66 (t, $^3J_{HH}$ = 7.6, 2H, 5-biph), 6.56 (vbr, fwhm = 22.5 Hz, 8H, *o*-Ph), 6.50 (d, $^3J_{HH}$ = 7.4, 3-biph).

^{13}C{^{1}H} NMR (126 MHz, CD_2Cl_2, 298 K): δ 162.3 (q, $^1J_{CB}$ = 50, [B(3,5-(CF_3)$_2\underline{C}_6H_3$)$_4$]$^-$), 152.5 (br d, $^1J_{RhC}$ = 42, 1-biph), 150.0 (s, 2-biph), 137.4 (s, 3-Ar^F), 135.3 (s, [B(3,5-(CF_3)$_2\underline{C}_6H_3$)$_4$]$^-$), 134.1 (t, $^3J_{PC}$ = 3, 4-Ar^F), 133.5 (t, J_{PC} = 6, *o*-Ph), 133.1 (s, 5-Ar^F), 131.9 (s, *p*-Ph), 131.7 (obscured, 2-Ar^F), 129.9 (s, 6-biph), 129.4 (qq, $^2J_{FC}$ = 32, $^3J_{CB}$ = 3, [B(3,5-(CF_3)$_2\underline{C}_6H_3$)$_4$]$^-$), 129.1 (obscured m, 6-Ar^F), 128.9 (t, J_{PC} = 5, *m*-Ph), 126.7 (t, J_{PC} = 19, 1-Ar^F), 126.1 (s, 5-biph), 125.9 (q, $^1J_{FC}$ = 276, CF_3), 125.2 (q, $^1J_{FC}$ = 272, [B(3,5-($\underline{C}F_3$)$_2C_6H_3$)$_4$]$^-$), 125.0 (obscured, *i*-Ph), 124.9 (s, 4-biph), 123.7 (s, 3-biph), 118.0 (sept, $^3J_{FC}$ = 4, [B(3,5-(CF_3)$_2\underline{C}_6H_3$)$_4$]$^-$).

^{31}P{^{1}H} NMR (162 MHz, CD_2Cl_2, 298 K): δ 20.1 (dh, $^1J_{RhP}$ = 124, $^2J_{PF}$ = 5).

^{19}F{^{1}H} NMR (376 MHz, CD_2Cl_2, 298 K): δ -62.89 (s, [B(3,5-(CF_3)$_2C_6H_3$)$_4$]$^-$), -66.39 (app. q, $^1J_{RhF} \approx {}^2J_{RhP}$ = 5, Ar^F).

HR ESI-MS (positive ion, 4 kV): 915.1222 ([M]$^+$, calcd 915.1246) *m/z*.

Anal. Calcd for $C_{82}H_{48}BF_{30}P_2Rh$ (1778.90 g·mol^{-1}): C, 55.37; H, 2.72; N, 0.00. Found: C, 55.56; H, 2.62; N, 0.0.

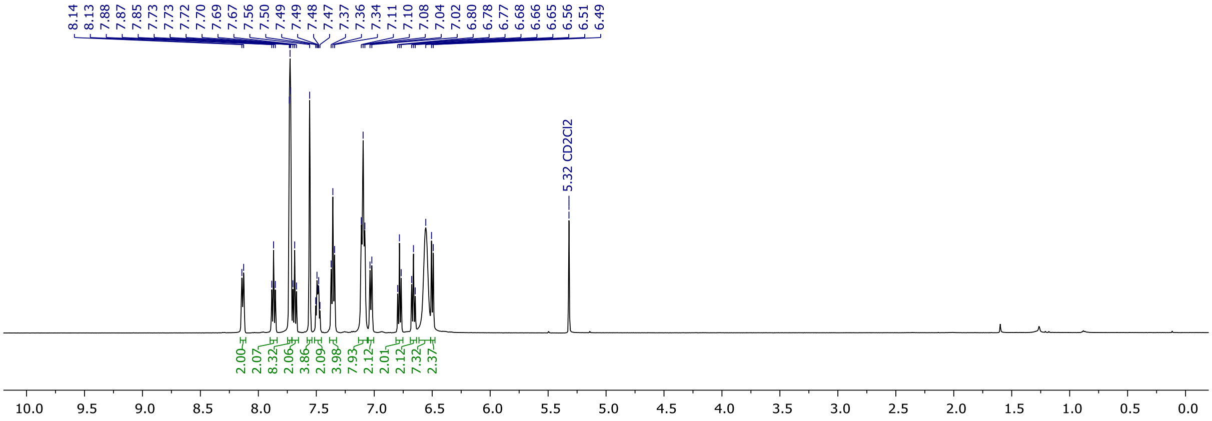

Figure S22: ^{1}H NMR spectrum of [Rh(biph)(PPh_2Ar^F)$_2$][BAr^F_4] **4** (500 MHz, CD_2Cl_2, 298 K).

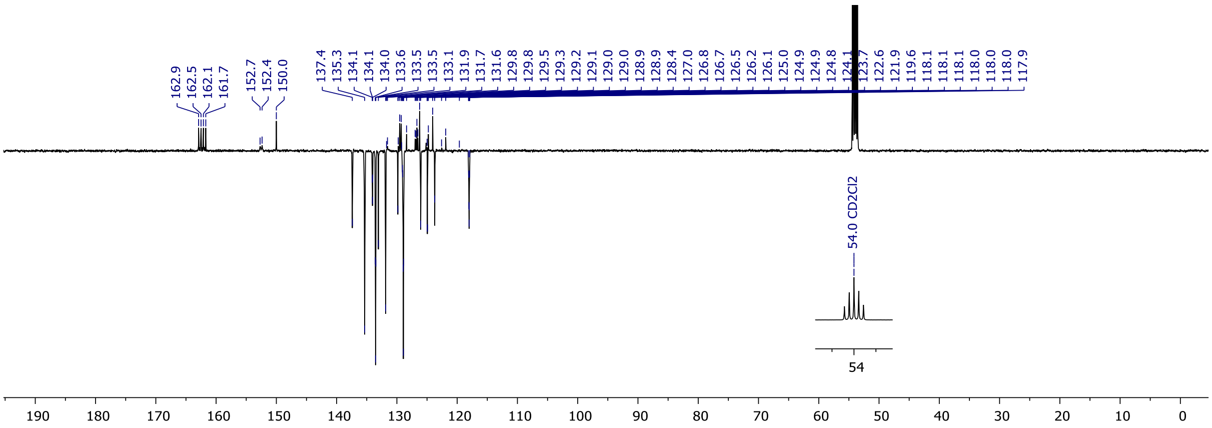

Figure 23: $^{13}C\{^{1}H\}$ APT NMR spectrum of [Rh(biph)(PPh_2Ar^F)$_2$][BAr^F_4] **4** (126 MHz, CD_2Cl_2, 298 K).

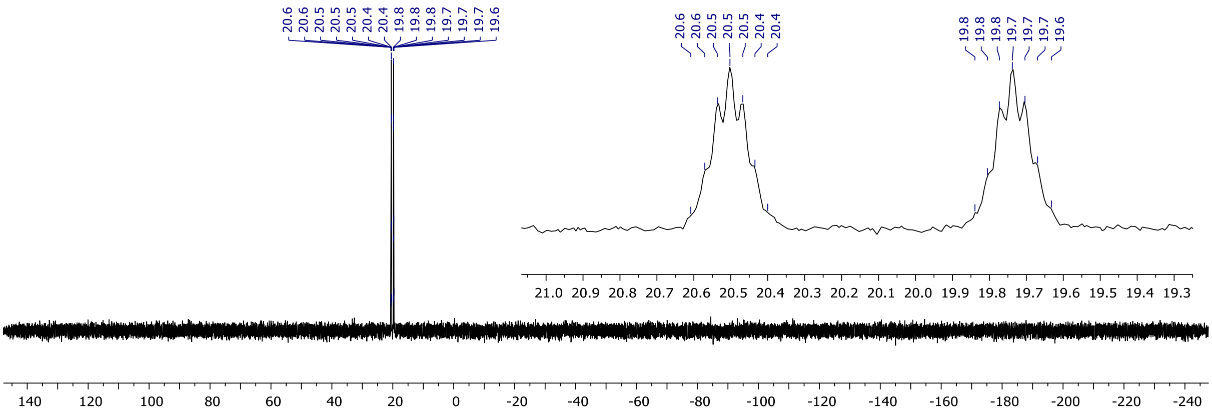

Figure S24: $^{31}P\{^{1}H\}$ NMR spectrum of [Rh(biph)(PPh_2Ar^F)$_2$][BAr^F_4] **4** (162 MHz, CD_2Cl_2, 298 K).

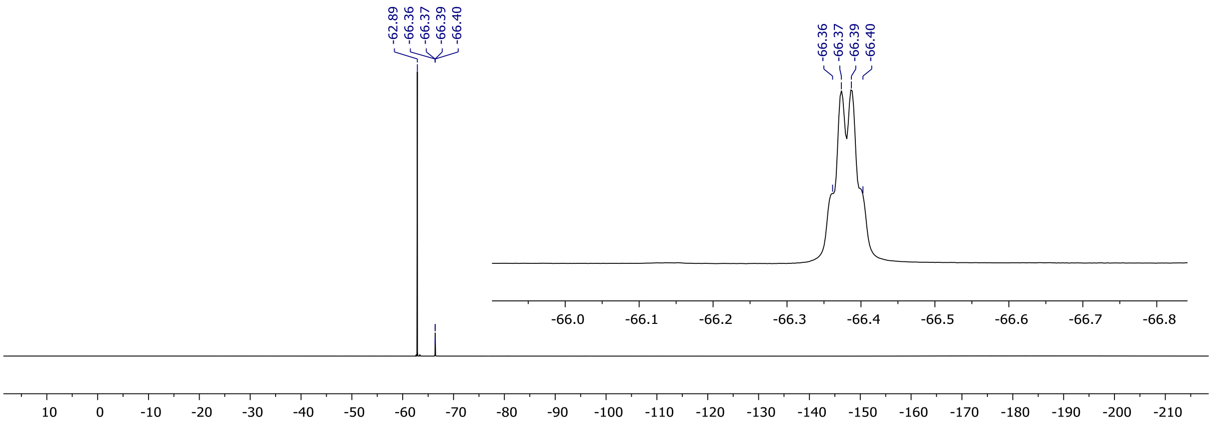

Figure S25: $^{19}F\{^{1}H\}$ NMR spectrum of [Rh(biph)(PPh_2Ar^F)$_2$][BAr^F_4] **4** (376 MHz, CD_2Cl_2, 298 K).

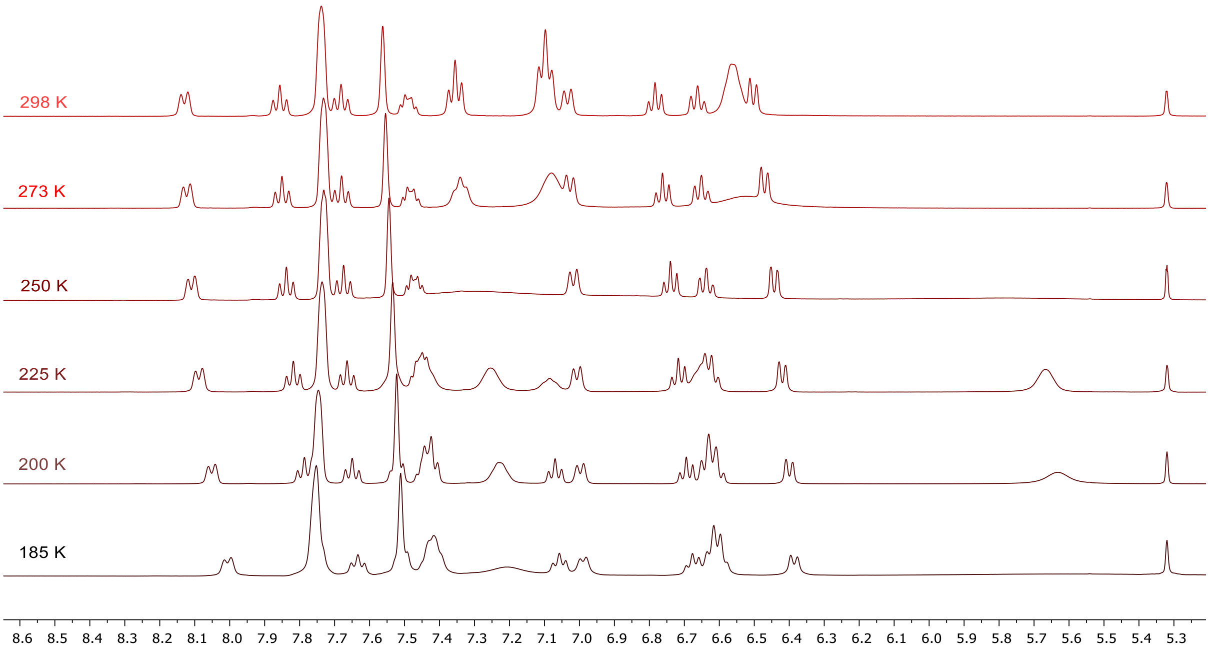

Figure S26: VT ^{1}H NMR spectra of [Rh(biph)(PPh_2Ar^F)$_2$][BAr^F_4] **4** (400 MHz, CD_2Cl_2).

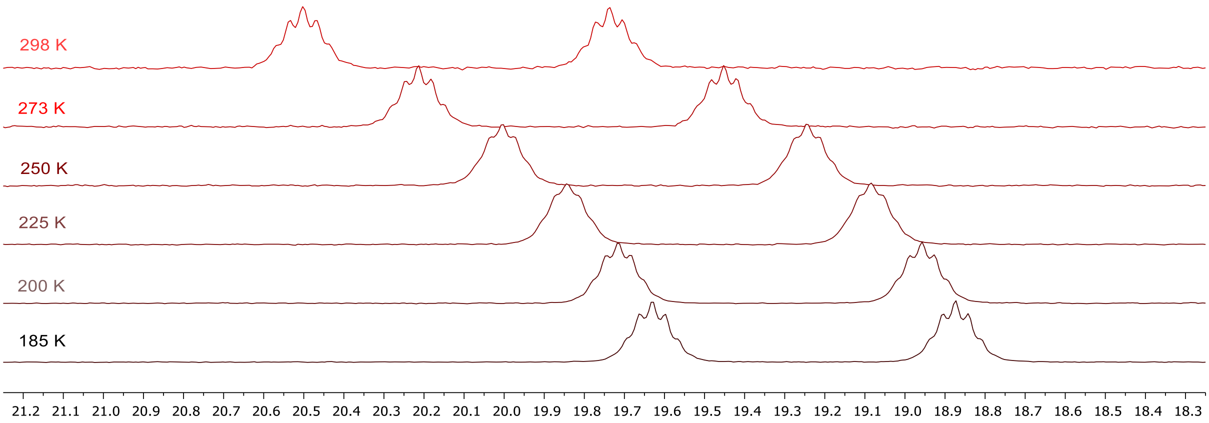

Figure S27: VT ^{31}P{^{1}H} NMR spectra of [Rh(biph)(PPh_2Ar^F)$_2$][BAr^F_4] **4** (162 MHz, CD_2Cl_2).

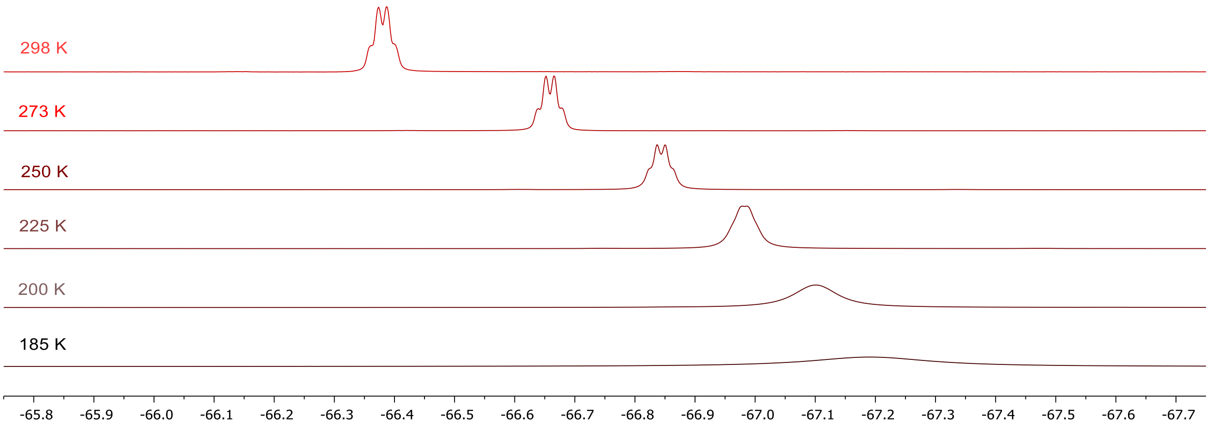

Figure S28: VT ^{19}F{^{1}H} NMR spectra of [Rh(biph)(PPh_2Ar^F)$_2$][BAr^F_4] **4** (376 MHz, CD_2Cl_2).

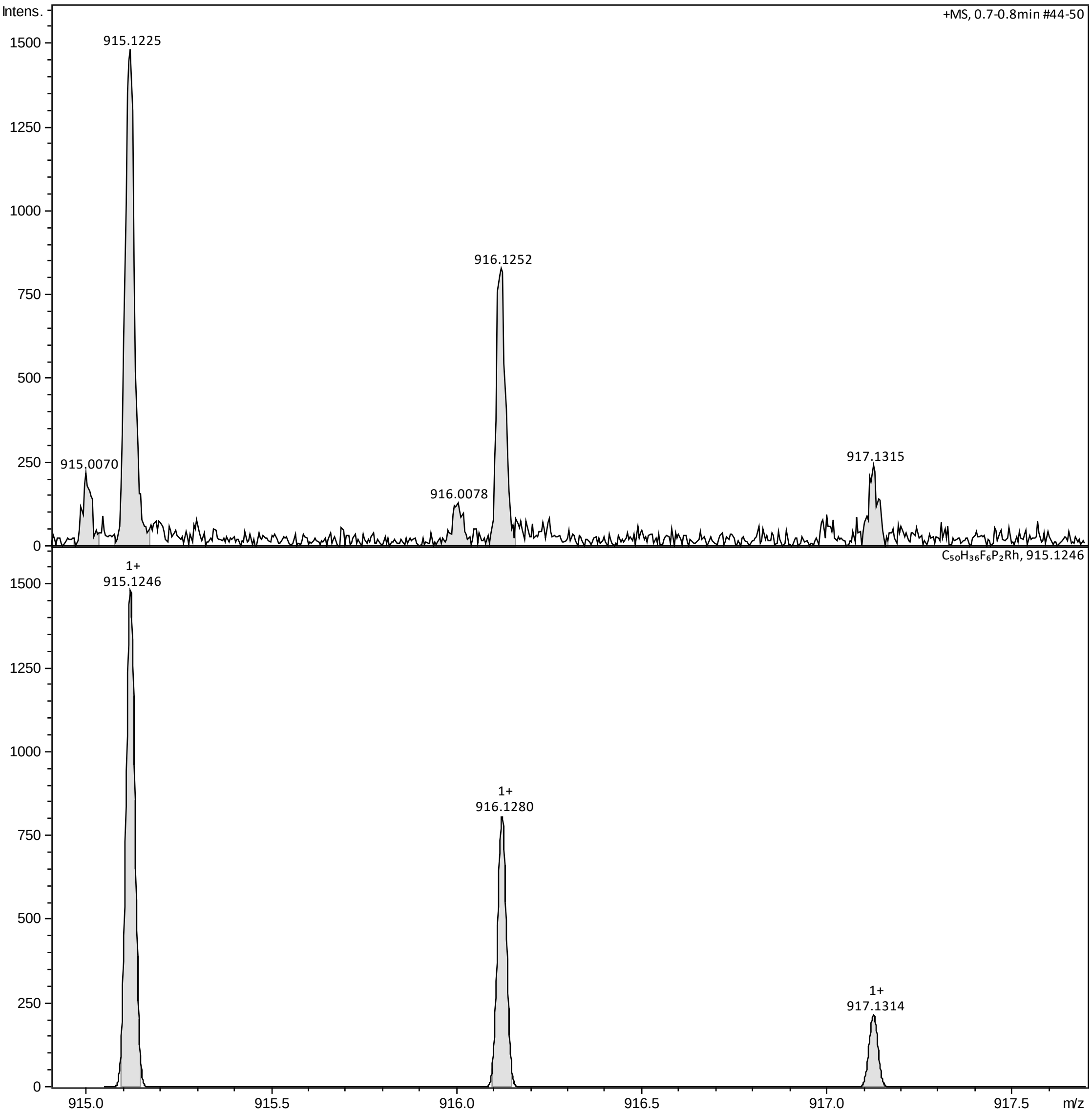

Figure S29: HR ESI-MS spectrum of $[Rh(biph)(PPh_2Ar^F)_2][BAr^F_4]$ **4**.

6. Preparation of [Rh(biph)(Cp)(PPh_2Ar^F)] 5

A suspension of **1** (62.1 mg, 50.0 µmol), Na[Cp] (9.7 mg, 110 µmol) in CH_2Cl_2 (5 mL) was stirred at ambient temperature for 18 h. The resulting pale-yellow solution was filtered, and the product crystallised by the addition of excess hexane (*ca*. 20 mL). Yield: 9.1 mg (13%, yellow solid).

^{1}H NMR (500 MHz, CD_2Cl_2, 298 K): δ 9.09 (dd, $^3J_{PH}$ = 16.3, $^3J_{HH}$ = 7.8, 1H, 6-Ar^F), 7.89 (t, $^3J_{HH}$ = 7.7, 1H, 5-Ar^F), 7.71 (t, $^3J_{HH}$ = 7.8, 1H, 4-Ar^F), 7.64 (d, $^3J_{HH}$ = 8.0, 1H, 3-Ar^F), 7.59 (d, $^3J_{HH}$ = 7.5, 2H, 6-biph), 7.19 (td, $^3J_{HH}$ = 7.5, $^5J_{PH}$ = 1.8, 2H, *p*-Ph), 7.02 (td, $^3J_{HH}$ = 7.8, $^4J_{PH}$ = 2.5, 4H, *m*-Ph), 6.95 (dd, $^3J_{HH}$ = 7.4, $^4J_{HH}$ = 1.5, 2H, 3-biph), 6.86 (dd, $^3J_{HH}$ = 8.0, $^3J_{PH}$ = 11.2, 4H, *o*-Ph), 6.77 (t, $^3J_{HH}$ = 7.4, 2H, 4-biph), 6.67 (td, $^3J_{HH}$ = 7.8, $^4J_{HH}$ = 2.5, 2H, 5-biph), 5.22 (s, 5H, Cp).

^{13}C{^{1}H} NMR (126 MHz, CD_2Cl_2, 298 K): δ 162.8 (dd, $^1J_{RhC}$ = 35, $^2J_{PC}$ = 17, 1-biph), 155.3 (s, 1-biph), 144.2 (d, $^2J_{PC}$ = 25, 6-Ar^F), 141.3 (d, $^3J_{RhC}$ = 2, 6-biph), 134.9 (d, $^1J_{PC}$ = 50, *i*-Ph), 132.7 (q, $^2J_{FC}$ = 33, 2-Ar^F), 132.0 (d, $^4J_{PC}$ = 2, 4-Ar^F), 131.6 (d, $^2J_{PC}$ = 10, *o*-Ph), 131.3 (d, $^3J_{PC}$ = 14, 5-Ar^F), 130.3 (d, $^1J_{PC}$ = 38, 1-Ar^F), 129.4 (obscured, 3-Ar^F), 129.4 (d, $^4J_{PC}$ = 3, *p*-Ph), 127.4 (d, $^3J_{PC}$ = 10, *m*-Ph), 124.0 (s, 5-biph), 123.8 (q, $^1J_{FC}$ = 274, CF_3), 122.7 (s, 4-biph), 121.9 (s, 3-biph), 93.1 (app. t, *J* = 3, Cp).

^{31}P{^{1}H} NMR (162 MHz, CD_2Cl_2, 298 K): δ 55.9 (d, $^1J_{RhP}$ = 167).

^{19}F{^{1}H} NMR (376 MHz, CD_2Cl_2, 298 K): δ -55.12 (s).

HR ESI-MS (positive ion, 4 kV): 650.0847 ($[M]^{+\cdot}$, calcd 684.0852) *m/z*.

Anal. Calcd for $C_{36}H_{27}F_3PRh$ (650.49 g·mol^{-1}): C, 66.47; H, 4.18; N, 0.00. Found: C, 66.39; H, 4.35; N, 0.00.

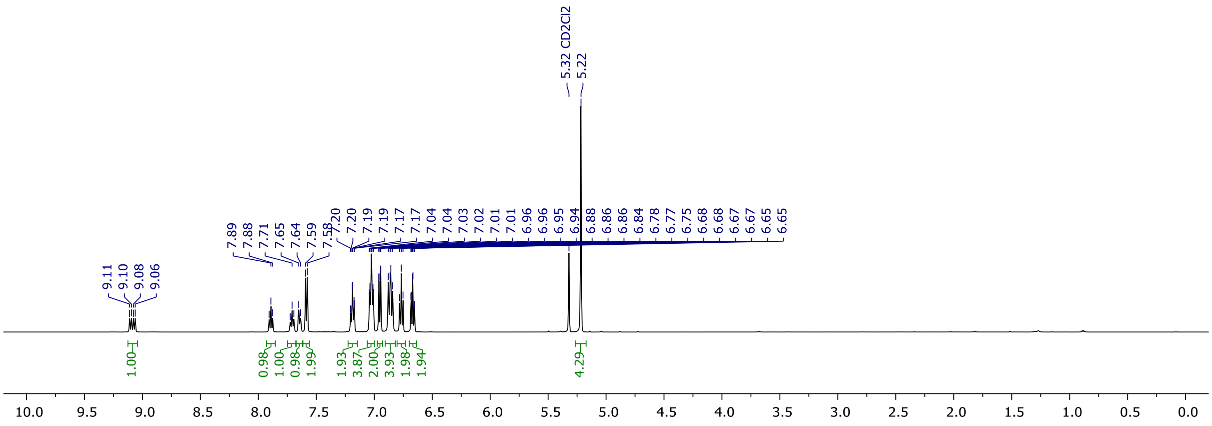

Figure S30: ^{1}H NMR spectrum of [Rh(biph)(Cp)(PPh_2Ar^F)] **5** (500 MHz, CD_2Cl_2, 298 K).

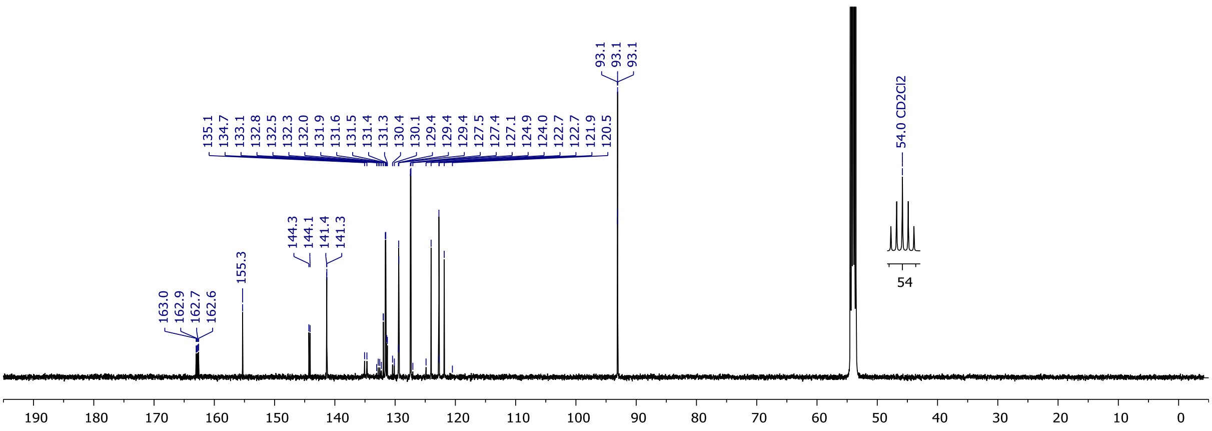

Figure S31: $^{13}C\{^{1}H\}$ NMR spectrum of [Rh(biph)(Cp)(PPh_2Ar^F)] **5** (126 MHz ,CD_2Cl_2, 298 K).

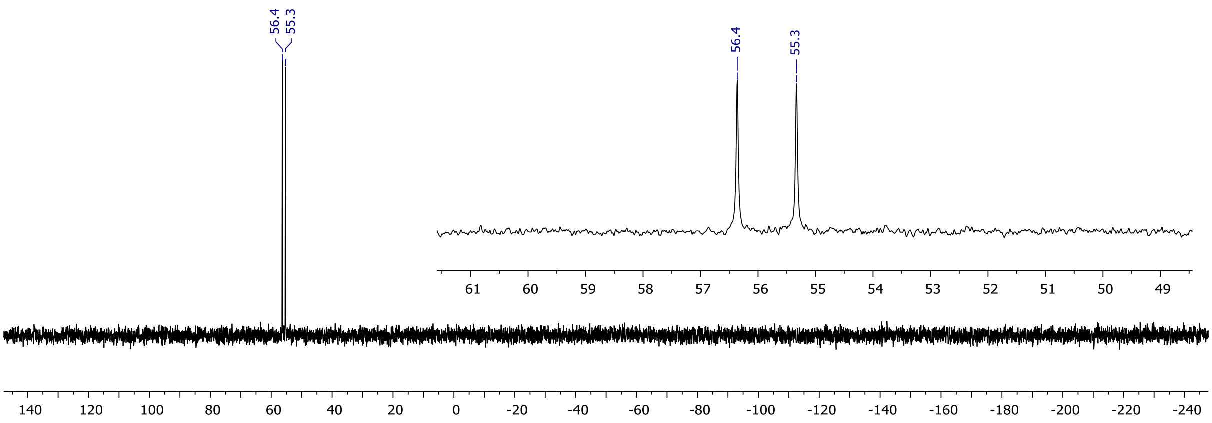

Figure S32: $^{31}P\{^{1}H\}$ NMR spectrum of [Rh(biph)(Cp)(PPh_2Ar^F)] **5** (162 MHz, CD_2Cl_2, 298 K).

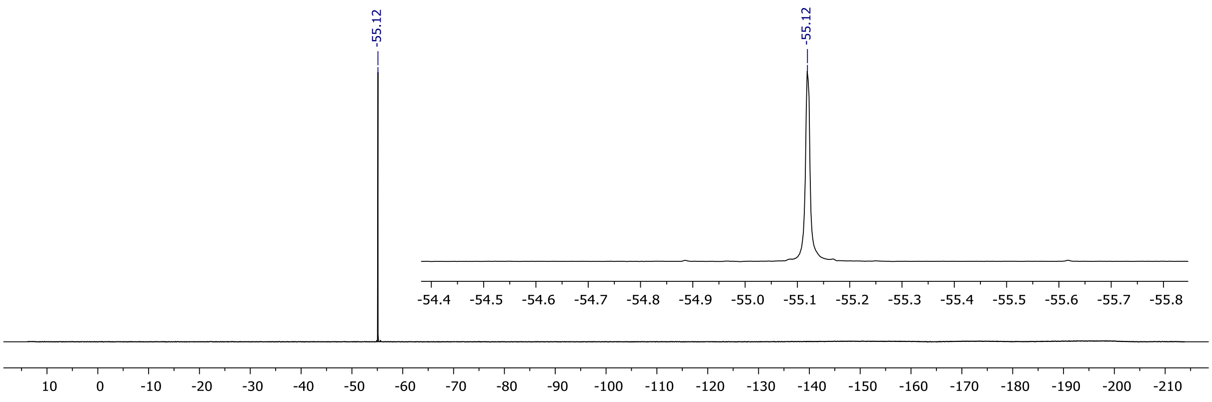

Figure S33: $^{19}F\{^{1}H\}$ NMR spectrum of [Rh(biph)(Cp)(PPh_2Ar^F)] **5** (376 MHz, CD_2Cl_2, 298 K).

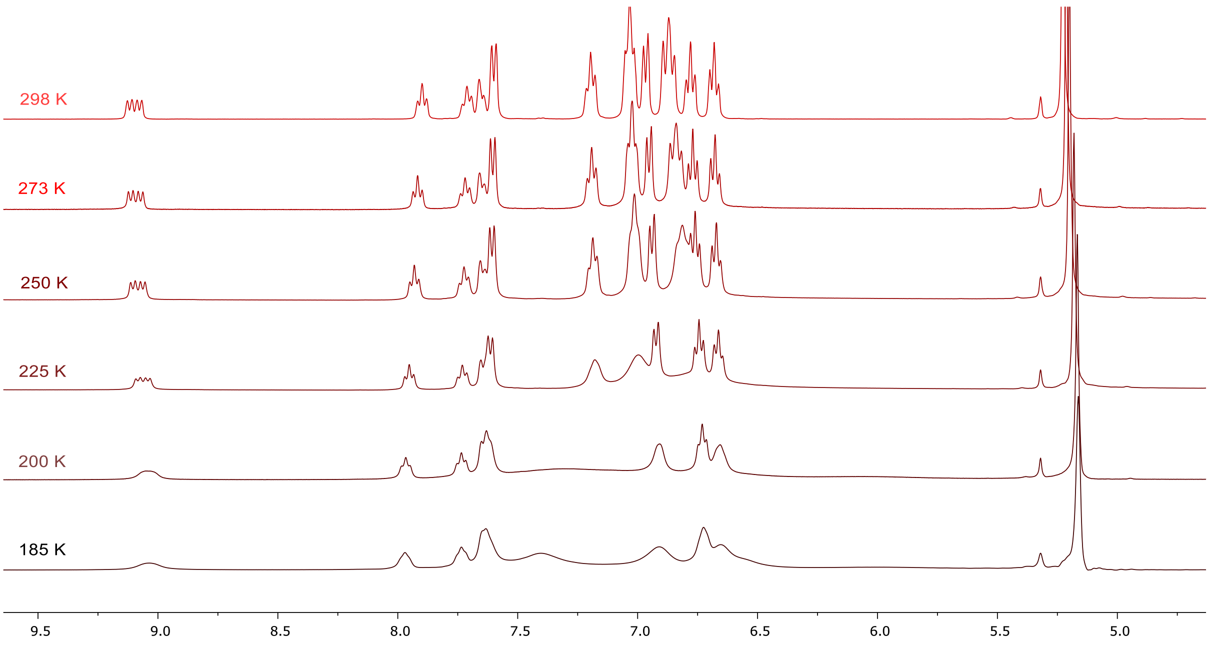

Figure S34: VT ^{1}H NMR spectra of [Rh(biph)(Cp)(PPh_2Ar^F)] **5** (400 MHz, CD_2Cl_2).

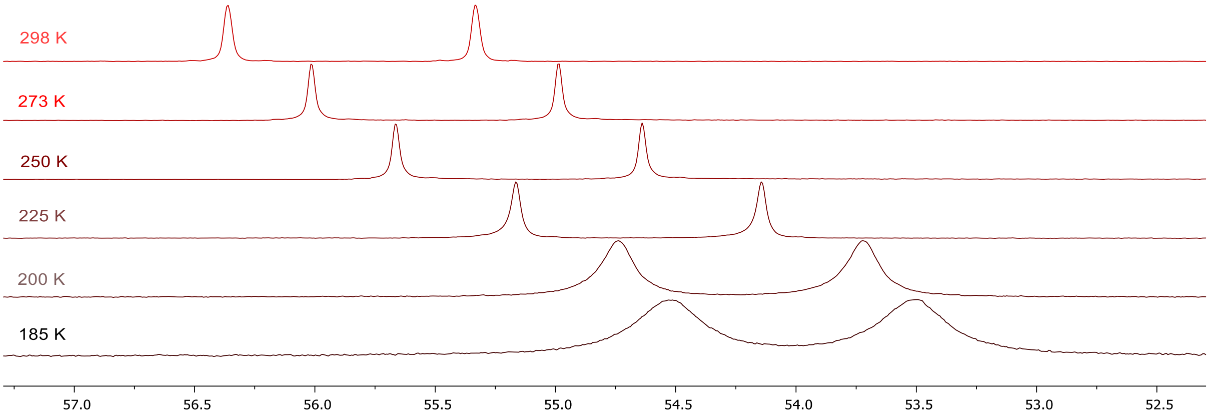

Figure S35: VT $^{31}P\{^{1}H\}$ NMR spectra of [Rh(biph)(Cp)(PPh_2Ar^F)] **5** (162 MHz, CD_2Cl_2).

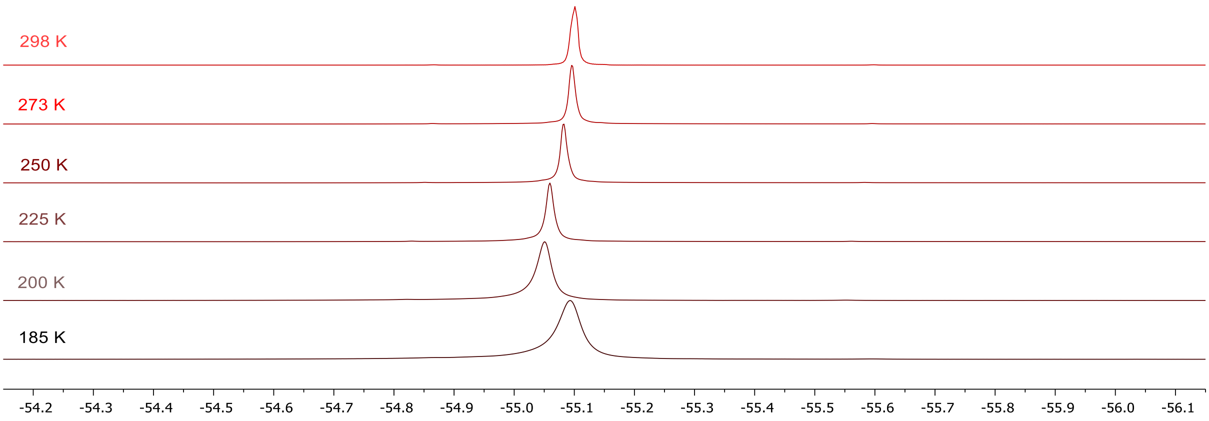

Figure S36: VT $^{19}F\{^{1}H\}$ NMR spectra of [Rh(biph)(Cp)(PPh_2Ar^F)] **5** (376 MHz, CD_2Cl_2).

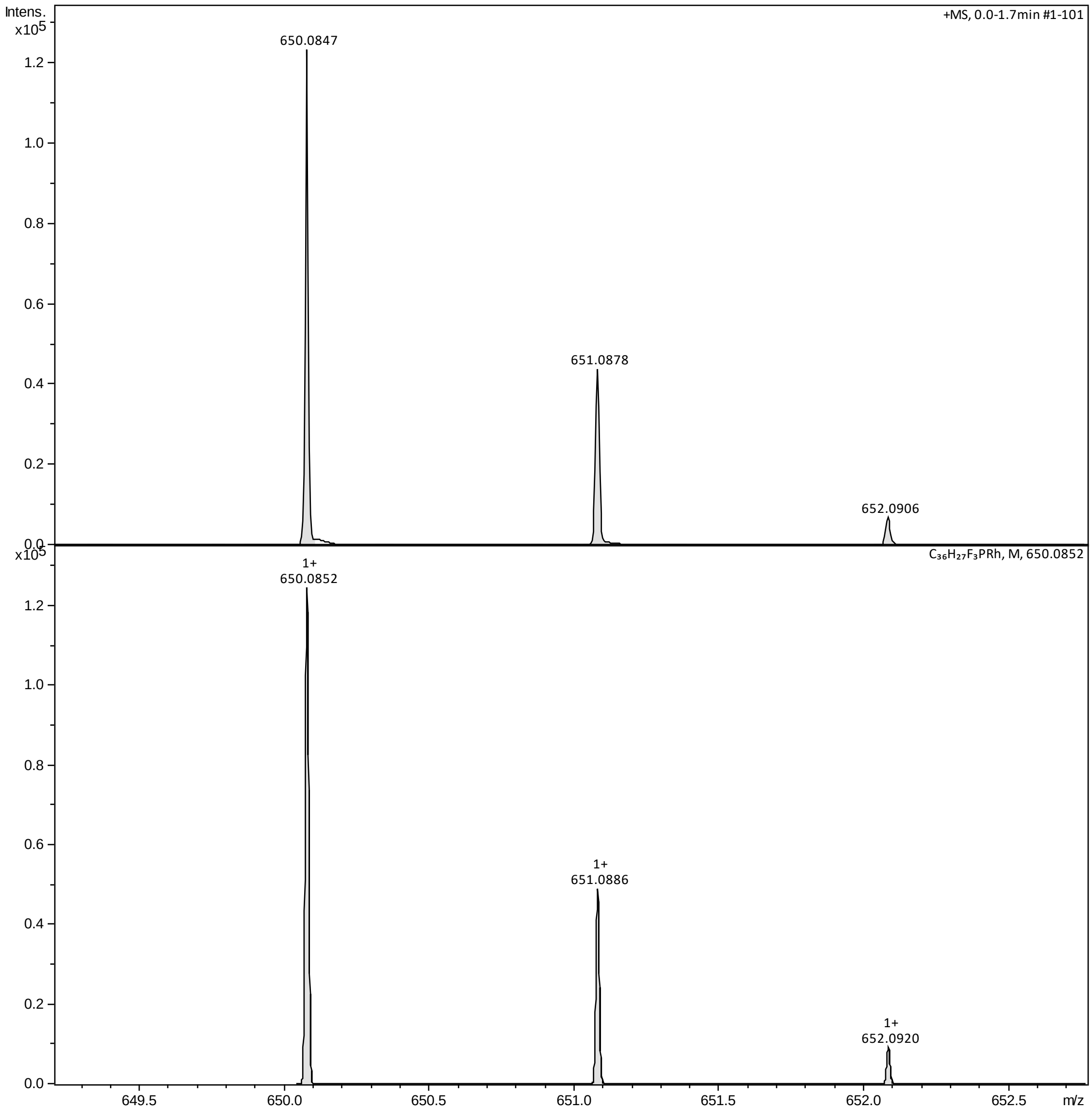

Figure S37: HR ESI-MS spectrum of [Rh(biph)(Cp)(PPh_2Ar^F)] **5**.

7. Line shape analysis

Approximate enthalpies of activation for the C–CF_3 restricted rotation observed by $^{19}F\{^1H\}$ NMR spectroscopy for **2** – **5** were determined by a pseudo Eyring analysis. These processes were treated as fast 1:1:1 three site exchanges, with the rate proportional to k' = 1/(W* - W_0); where W* is the linewidth of the exchanging resonance and W_0 is the linewidth in the absence of exchange.[6] The activation enthalpies were correspondingly extracted from a plot of ln(k'/T) vs 1/T (Figure S38), exploiting logarithmic identities, using selected low temperature data. Linewidths were determined using MestReNova (9.0), with the value of W_0 set at 2.5 Hz on the basis of data for the $[BAr^F{}_4]^-$ counter anion at high temperature.

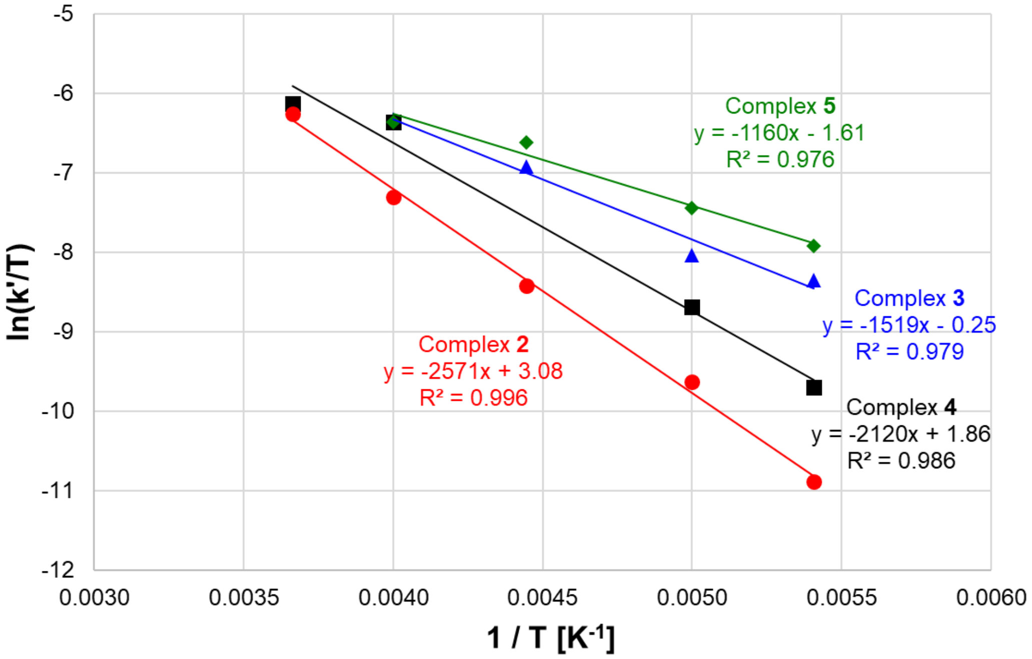

Figure S38: Pseudo Eyring Plot for the restricted C–CF_3 bond rotation observed in **2** – **5**.

8. References

1 C. N. Iverson, W. D. Jones, *Organometallics* **2001**, *20*, 5745–5750.

2 D. Peral, D. Herrera, J. Real, T. Flor, J. C. Bayón, *Catal. Sci. Technol.* **2016**, *6*, 800–808.

3 P. Schroll, B. König, *Eur. J. Org. Chem.* **2015**, 309–313.

4 T. K. Panda, M. T. Gamer, P. W. Roesky, *Organometallics* **2003**, *22*, 877–878.

5 W. E. Buschmann, J. S. Miller, K. Bowman-James, C. N. Miller, *Inorg. Synth.* **2002**, *33*, 83–91.

6 (a) J. W. Akitt, B. E. Mann, *NMR and Chemistry: An introduction to modern NMR spectroscopy*, Stanley Thornes, Cheltenham, **2000**; (b) J. Sandström, *Dynamic NMR spectroscopy*, Academic Press, London, **1982**.
